# Insights into the Sesquiterpenoid Pathway by Metabolic Profiling and *De novo* Transcriptome Assembly of Stem-Chicory (*Cichorium intybus* Cultigroup “Catalogna”)

**DOI:** 10.3389/fpls.2016.01676

**Published:** 2016-11-08

**Authors:** Giulio Testone, Giovanni Mele, Elisabetta Di Giacomo, Maria Gonnella, Massimiliano Renna, Gian Carlo Tenore, Chiara Nicolodi, Giovanna Frugis, Maria Adelaide Iannelli, Giuseppe Arnesi, Alessandro Schiappa, Donato Giannino

**Affiliations:** ^1^Institute of Agricultural Biology and Biotechnology, National Research CouncilRome, Italy; ^2^Institute of Sciences of Food Production, National Research CouncilBari, Italy; ^3^Department of Agricultural and Environmental Science, University of BariBari, Italy; ^4^Department of Pharmacy, University of Naples Federico IINaples, Italy; ^5^Enza Zaden ItaliaTarquinia, Italy

**Keywords:** *Cichorium intybus*, stem-chicory landraces, transcriptome, sesquiterpene lactones, bitterness

## Abstract

Stem-chicory of the “Catalogna” group is a vegetable consumed for bitter-flavored stems. Type and levels of bitter sesquiterpene lactones (STLs) participate in conferring bitterness in vegetables. The content of lactucin—and lactucopocrin-like STLs was higher in “Molfettese” than “Galatina” landrace stalks, regardless of the cultivation sites, consistently with bitterness scores and gustative differences. The “Galatina” transcriptome assembly resulted in 58,872 unigenes, 77% of which were annotated, paving the way to molecular investigation of the STL pathway. Comparative transcriptome analysis allowed the identification of 69,352 SNPs and of 1640 differentially expressed genes that maintained the pattern independently of the site. Enrichment analyses revealed that 4 out of 29 unigenes were up-regulated in “Molfettese” vs “Galatina” within the sesquiterpenoid pathway. The expression of two *germacrene A* -*synthase* (*GAS*) and one -*oxidase* (*GAO*) genes of the costunolide branch correlated positively with the contents of lactucin-like molecules, supporting that STL biosynthesis regulation occurs at the transcriptional level. Finally, 46 genes encoding transcription factors (*TFs*) maintained a differential expression pattern between the two varieties regardless of the growth site; correlation analyses among *TFs, GAS, GAO* gene expressions and STLs contents suggest that one *MYB* and one *bHLH* may act in the pathway.

## Introduction

Chicory (*Cichorium intybus* L.) is cultivated worldwide to produce food, coffee surrogates, forages pharmaceuticals, and healthcare compounds (Street et al., 2013). Genetic diversity analysis supported the three-cluster structure of *C. intybus* cultivated germplasm (Kiers et al., 2000; Raulier et al., 2016): witloof, root and leaf chicory groups. The latter embraces “Radicchio,” “Sugarloaf” and “Catalogna” sub-groups. Several “Catalogna” cultivars/landraces are cultivated in Italy for both leaves and stems. These latter are appreciated for the bitter and crispy taste. Botanically, they bear a receptacle made of outer whorls of leaves (runcinated-pinnatifid type, large mid-rib) and an inner bulk of inflorescence stems (syn. flower stalks, turions). These stalks (cut at various lengths) are mostly eaten raw (sliced into curly and crunchy strips, a.k.a. “puntarelle”) or cooked. Stem vegetables are novel products moving from a niche to a global market, showing potential use in the minimally or fully processed food chain (Renna et al., 2014).

The *C. intybus* species has a large (2n = 2x = 18; size 1405 Mbp) and complex genome (De Simone et al., 1997; Berardes et al., 2013). Culti-groups are mostly allogamous due to different mechanisms of self-incompatibility (Eenink, 1981, 1982; Varotto et al., 1995) and natural hybrids widely occur (Kiaer et al., 2009; Bai et al., 2012). Consistently, the “Catalogna” sub-group shows a high genetic variation at both inter- and intra- population levels (Raulier et al., 2016). So far, the *C. intybus* genetic toolbox includes a linkage consensus map (Cadalen et al., 2010), BAC libraries (Gonthier et al., 2010), EST databases (The Compositae Genome Project, 2000; Legrand et al., 2007; Dauchot et al., 2009), and transcriptomes (Hodgins et al., 2014). An increasing number of tools for molecular marker assisted breeding is predicted for the improvement of a wide range of chicory products.

Sesquiterpene lactones (STL) are secondary metabolites typical of *Asteraceae* spp., concentrated in the latex (Sessa et al., 2000) and active in defense against pathogens (Peña-Espinoza et al., 2015). From a nutritional standpoint, STL have both beneficial (e.g., anti-cancer, anti-leukemic) and allergenic properties (Chadwick et al., 2013), they contribute to the bitter taste of chicory food (Price et al., 1990; van Beek et al., 1990) and the contents vary significantly among culti-groups (Ferioli et al., 2015; Graziani et al., 2015). Bitterness is crucial for vegetable quality as high levels can cause rejection (D'Antuono et al., 2016), though the acceptance varies with consumers' use and culture (Drewnowski and Gomez-Carneros, 2000). Chemically, STL are C-15 terpenoids based on a guaiane skeleton bearing lactone rings (Cordell, 1976; Chadwick et al., 2013). The most abundant STLs of chicory leaves are lactucin, 8-deoxylactucin, lactucopicrin and the respective 11(S), 13-dihydroderivatives (Ferioli et al., 2015); glycosyl- and oxalyl- conjugate forms also occur (Kisiel and Zielinska, 2001; Graziani et al., 2015). As for STL biosynthesis, the enzymes germacrene A-synthase, -oxidase and costunolide synthase act upstream the pathway to convert farnesyl diphosphate into costunolide, this latter being the common precursor of STLs (de Kraker et al., 2002). The enzymes and respective genes (*GAS, germacrene A synthase; GAO, germacrene A oxidase;* COS, *costunolide synthase*) were characterized in several *Asteraceae* species (Nguyen et al., 2010; Cankar et al., 2011; Ikezawa et al., 2011; Liu et al., 2011; Ramirez et al., 2013; Eljounaidi et al., 2014, 2015). Terpene synthase genes of the STL pathway have been identified in model plants (Kitaoka et al., 2015; Tholl, 2015) and crops, such as tomato (Falara et al., 2011) and grapevine (Schwab and Wust, 2015). However, to date, enzymes and genes leading to the Lc- and Lp-compounds have not been identified yet.

A major aim of this work was to characterize the factors that contribute to bitterness at the metabolic and genetic levels by comparing “Galatina” (Gal) and “Molfettese” (Mol) landraces. The STL quantification revealed a higher content of lactucins and lactupicrins in the latter compared to the former, independently of the growing sites. Due to the lack of comprehensive genomic information on *C. intybus*, a reference Gal transcriptome for the “Catalogna” group was created. Digital gene expression (DGE) targeted to stems at the commercial maturity revealed those differentially expressed genes (DEGs), which maintained the patterns in Mol and Gal landraces irrespective of the cultivation area. Focusing on transcriptomic differences of the STL pathway, the Mol genotype was enriched of upregulated genes—two *germacrene A-synthase* and one *oxidase* (*GAS* and *GAO*)—acting upstream the route. The *GAS* and *GAO* transcription levels correlated positively with the contents of 11(S), 13-dihydrolactucin and 11(s), 13-dihydro-8-deoxylactucin, supporting that STL biosynthesis regulation occurs at the transcriptional level. Consequently, the *GAS* and *GAO* higher expression levels of Mol vs. Gal may account for higher contents of STLs, supporting that these genes may be good expression markers for bitterness selection. Correlation analyses among the expression levels of *GAS, GAO*, and transcription factor (TF) genes and STLs contents pointed at MYB and bHLH as the best TF candidates in the STL biosynthesis regulation.

## Materials and methods

### Plant materials and sampling

The “Galatina” (Gal) and “Molfettese” (Mol) landraces of stem chicory (*Cichorium intybus* L. “Catalogna” group) were previously described, including botanical classification, phenotypical traits, site coordinates, and cultivation parameters (D'Acunzo et al., 2016). In the current work, the landraces were grown both on local private farms in Apulia (Molfetta, southern Italy) and in the Enza Zaden fields (Tarquinia, Viterbo, Lazio, Italy). Plants were sown in August 2012 in both locations and transplanted after 30 days. Plant density was 8.3 plants/m^2^ and similar growing techniques were applied in both growing areas. As for Lazio, harvesting was on the 08/01/2013 and 28/01/2013 for Mol and Gal, respectively; the average temperatures 1 week before harvesting were 9.0 ± 0.9°C and 6.9 ± 1.4°C and the average temperature during the 1–28/1/2013 period was 8.2 ± 2.2°C (www.idrografico.roma.it/annali). As for Apulia, harvesting occurred on the 14/01/2013 and 25/01/2013 for Mol and Gal, respectively; the average temperatures 1 week before harvesting were 9.4 ± 0.1°C and 9.5 ± 0.3°C, and the average temperature of the 1–25/1/2013 lapse was 9.7 ± 0.2°C (www.agrometeopuglia.it). Harvested plants were brought to laboratories, selected for comparable weights (Lazio: 905 ± 230gr and 850 ± 201gr for Gal and Mol; Apulia: 820 ± 162gr and 940 ± 128gr for Gal and Mol; non-significant differences were scored by ANOVA) and processed. In the experiments of STL lactones quantification, transcriptional and allelic variation analyses, marketable stems were removed from rosettes (*n* = 15) of each landrace. A replicate batch consisted of 10 homogeneous stems (mean length 11.5 ± 1.5 cm, mean diameter of the median section: 2.7 ± 0.3 cm). These were immediately frozen in liquid nitrogen and stored at −80°C for RNA isolation or they were lyophilized at −50°C for 72 h (laboratory freeze dryer with stoppering tray dryer, FreeZone®, Labconco Corp., Kansas City, MO, USA) and stored at −20°C for HPLC analyses. Three biological replicates were used in all the experiments.

### Sesquiterpene lactones quantification

In order to quantify the total amount of STL (free and bound fractions), the samples were prepared by adopting both cellulase hydrolytic treatment (Tamaki et al., 1995) and ultrasound assisted extraction (UAE). As regards the enzymatic procedure, the lyophilized sample (2 g) was added to 50 mL of methanol/water solution (80:20, v/v) plus 2% of formic acid and 3 mL of santonin solution (101.7 μg/mL) as internal standard, and shaken (1000 g/min, for 15 min, at 80 °C; F80 Digit, Falc Instruments s.r.l., Italy). The supernatant was collected and the pellet underwent two additional extractions as described above. The final extract (about 150 mL) was dried under vacuum, re-dissolved in methanol/dichloromethane (1:7, v/v) solution, and loaded onto a solid phase extraction (SPE) column. The elution was carried out with 6 mL of a dichloromethane/ethyl acetate (3:2 v/v) solution; the eluted fractions were pooled and then vacuum-dried. The eluted fraction was re-dissolved in 1 mL of a cellulase enzyme solution (10 mg of cellulase/mL of water) and then incubated at 37°C for 2 h with stirring. The solvent was evaporated and then made up to 500 μL. As for the ultrasound assisted extraction, the same protocol as described above was adopted. Differently, the SPE eluted fraction was sonicated at 50 KHz for 30 min (37°C) by using an ultrasound bath (Labsonic LBS1-3, Falc Instruments s.r.l., Italy). The purified samples were added with methanol (4 mL) and the STL identification was performed by an HPLC apparatus Finnigan (Thermo Electron Corporation, San Jose, California), equipped with quaternary pump, DAD detector, and a C18 Kinetex column (250 × 4.60 mm, 5 μm). The mobile phases A and B were, respectively, methanol/water 14:86 and 64:36 (v/v). The gradients were 0–20 min, 100–58% A; 20–30 min, 58% A; 30–45 min, 58–0% A; 45–50 min, 0% A; 50–52 min, 0–100% A; 52–62 min, 100% A. The flow was at 0.5 mL/min and the injection volume was 80 μL. STL peaks were monitored at 260 nm. Following an identical preliminary extraction protocol, UAE lead to equivalent amounts of STL as compared to the cellulase treatment (Table S1).

### RNA isolation and sequencing

For transcriptome reference assembly, Gal plants at the transplant (*n* = 5, 3–4 true leaves) and commercial maturation (*n* = 5) stages were used; distinct tissues (apices, stems, leaves and roots) were sampled and ground in liquid nitrogen. 500 mg of each tissues was used to isolate total RNA by TRIzol reagent (Invitrogen) followed by an additional purification step using RNAeasy separation columns (RNAeasy kit, Qiagen); yields were estimated by electrophoretic and spectrophotometric analyses (NanoDrop ND-1000; Thermo Scientific Inc.), and RNA integrity number (RIN > 7) was verified using BioAnalyzer 2100 (Agilent Technologies Inc). cDNA libraries were prepared using TruSeq RNA-seq sample preparation kit (Illumina) and sequenced in 100 bp paired-end mode using an Illumina HiSeq2000 (IGA Technology Services, Udine, Italy).

As for NGS transcriptional analyses and SNP mining, size-comparable stems (*n* = 10) at harvesting time were sampled from Gal (*n* = 5) and Mol (*n* = 5) grown in each location. Total RNA was isolated, quantified and controlled for yield and integrity as described above. Illumina Truseq cDNA libraries were prepared and sequenced in 50 bp single-end on Illumina HiSeq2000 platform. For a given genotype, three biological replicates for each growing area were generated. RNAseq data sets have been stored in the National Center for Biotechnology Information database (NCBI, www.ncbi.nlm.nih.gov) under the BioProject accession number PRJNA328202.

### Transcriptome assembly

Paired-end Illumina raw reads were filtered to remove adapter contaminations and low-quality reads using Trimmomatic v0.32 (Bolger et al., 2014). The high-quality reads were assembled by using both a one-step (*de novo*) and two-steps (EST-based backbone construction followed by a *de novo* assembly) approaches. In the one-step approach the cleaned reads were assembled using Trinity (Grabherr et al., 2011) with default parameters. In the two-steps approach, a collection of 53,973 *Cichorium intybus* EST was retrieved from the NCBI database. To create a unigene dataset, the EST were cleaned (trimming of vector tag, low quality stretches and repetitive element) and assembled using the EGassembler pipeline (Masoudi-Nejad et al., 2006) with the default parameters. The pipeline produced 26,085 unigenes (7199 contigs and 18,886 singletons). The filtered reads were mapped on the resulting unigenes by using Bowtie2 (Langmead and Salzberg, 2012). The unmapped reads were recovered and used in iterative contig extension processes using SeqMan Pro (DNASTAR. Madison, WI). The reads that did not extended contig lengths were *de novo* assembled Velvet/Oasis programs (Zerbino and Birney, 2008; Schulz et al., 2012) with a *k-mer* size of 25. Redundancy between one—and two-steps output was removed by TGICL-CAP3 (Pertea et al., 2003) using overlaping stretches 200 bp-long and minimal identity of 97%. The genes/isoforms clustering was performed using cd-hit-est from the CD-HIT package (Li and Godzik, 2006) with sequence identity threshold of 97%. The longest transcripts were selected as representative for each cluster.

### Functional annotation

Transcripts were annotated using BlastX (cut-off *E*-value of 10–5) mining the following databases: Nr, NCBI non-redundant database (January 12, 2015); TAIR, The Arabidopsis Information Resource (TAIR10); SwissProt and TrEMBL, the manually annotated and reviewed and the automatically annotated and not reviewed sections of the UniProt Knowledgebase (UniProtKB), respectively (release 2014_02); KOG, euKaryotic Ortholog Groups (Koonin et al., 2004). Blast2GO (Conesa et al., 2005) was used to obtain Gene Ontology (GO) and KEGG (Kanehisa and Goto, 2000) annotations based on BLASTx hits against the Nr database. WEGO (Ye et al., 2006) was used for GO functional classification. To improve the pathway annotation, unigenes were also submitted to the online KEGG Automatic Annotation Server (Moriya et al., 2007). Deduced protein sequences were analyzed with InterProScan 5.1–44.0 (Jones et al., 2014) against 15 integrated databases (Phobius, TMHMM, Pfam, ProDom, Gene3d, Panther, SuperFamily, Coils, SMART, PrositeProfiles, PRINTS, SignalP, PIRSF, TIGRFAMs, HAMAP) and protein signatures were collected.

### Polymorphisms calling

BWA (Li and Durbin, 2009), Picard tools (http://picard.sourceforge.net), SAMtools (Li et al., 2009) and the BCFtools utilities were used to align the reads of Gal and Mol to the reference transcriptome, mark duplicated reads, compute the genotype likelihoods and call the variable positions, respectively. In order to provide a set of reliable SNPs useful in robust genotyping assays, the following filtering criteria were imposed: (a) quality score (“QUAL”) ≥ 30 (99.9% base call accuracy); (b) at least 10 high-quality reads (“DP4”) supporting the nucleotide differences; (c) SNP within homopolymer stretches of length ≥ 5 bp were excluded; (d) genotype quality score (“GQ”) ≥ 50. The MISA perl script (http://pgrc.ipk-gatersleben.de/misa) was used for identification of potential simple sequence repeats (SSRs). Units with one to six nucleotides and a minimum repetition of twelve units for mono-nucleotides, six for di-nucleotides, five for tri-, tetra-, penta- and hexa-nucelotides were considered in the analysis.

### Gene expression analyses

The raw single-end reads were trimmed as described above. For a given genotype, the cleaned reads were mapped on the reference assembly using BWA (Li and Durbin, 2009) and SAMtool pipeline, and read count for each transcript was scored in each replicate. The digital gene expression (DGE) levels were calculated and expressed as RPKM (reads *per* kilobases of transcript sequence *per* million of mapped reads) values.

### Real time quantitative PCR (qPCR)

Total RNA derived from a pool (*n* = 5) of stems was isolated by the RNeasy Plant Mini Kit (Qiagen), DNase treated (RQ1, Promega), and 1 μg was reverse-transcribed at 55°C by SuperscriptIII (Life Technologies). The cDNA (100 ng) was amplified by Eco Real-Time PCR System (Illumina) using 1x Quantimix easy master mix (Biotools) and 0.3 μM of each primer in a 10 μl final volume. The triplicate reaction conditions were as follows: 95°C for 10 min, 45 cycles at 95°C for 15 s, 60°C for 15 s, and 72°C for 40 s. Primer specificity was checked by melting curve analysis and by agarose gel electrophoresis. Three technical replicates and three independent biological experiments were performed for each sample. The expression levels of the target unigenes were normalized with the reference genes *ACT* (Maroufi et al., 2010) by the Q-Gene program (Muller et al., 2002). Primers were designed using Primer3 software (Untergasser et al., 2012) and they are listed in Table S2.

### Statistical analyses

The analysis of variance (ANOVA) was applied to STL content variation in landraces grown in Apulia and Lazio cultivation sites, followed by Duncan Multiple Range Test. All procedures, General Linear Model and means separation, were carried out by Statistical Analysis System program (SAS software, Version 9.1, Cary, NC, USA). The principal component analysis (PCA) allowed a visual overview about spatial distribution and grouping among genotypes and growing sites; it was based on mean centered and standardized data (unit variance scaled) and results were shown as bi-plots of scores (treatments) and loadings (variables) plots (XLStat Pro, Addinsoft, Paris, France). Differential expression analysis was performed using the Bioconductor edgeR package (Robinson et al., 2010). All samples were normalized by trimmed mean of M values (TMM). Unigenes with at least 1 read per million in at least 3 samples were retained and a false discovery rate (FDR) value ≤ 0.05 and an absolute value of log_2_ fold change ratio (log_2_FC) ≥ 1 were set as the thresholds for the significance of the gene expression difference. The hypergeometric test (Pang et al., 2013) was used for GO and KEGG enrichment analyses of differentially expressed genes with the whole transcriptome set as background. The significant GO and KEGG terms were identified after multiple testing adjustments with an FDR ≤ 0.05. Correlations analyses were performed in R 3.2.2 (R Core Team, 2015) with the *rcorr* function of the *Hmisc* package (Harrell, 2016), whilst *corrplot* function (Wei, 2013) was used to produce the correlation matrix.

## Results

### Stems of “Molfettese” contain more STL than “Galatina” independently of cultivation area

**Figure 1:**
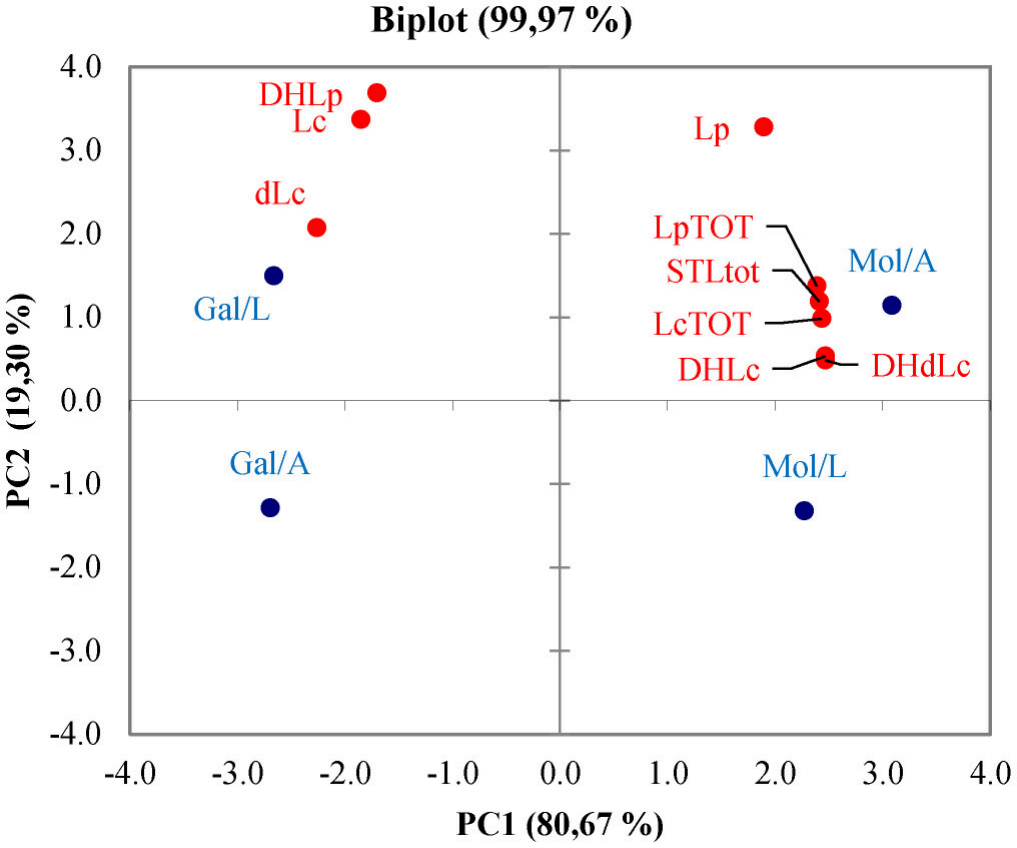
**Principal component analysis biplot showing the spatial distribution of the sesquiterpene lactones content in Mol and Gal puntarelle types cultivated in Lazio (L) and Apulia (A)**. Lc, lactucin; DHLc, 11(S), 13-dihydrolactucin; dLc, 8-deoxylactucin; DHdLc, 11(S), 13-dihydro-8-deoxylactucin; LcTOT, total of lactucin-like STLs; Lp, lactucopicrin; DHLp, 11(s), 13-dihydrolactucopicrin; LpTOT, total of lactucopicrin-like STLs; STLs, total STL content.

The major STLs lactucin (Lc), 8-deoxylactucin (dLc), lactucopicrin (Lp) and the respective dihydro-derivatives, 1,3-dihydrolactucin (DHLc), 11(s), 13-dihydro-8-deoxylactucin (DHdLc), 11(s), 13-dihydrolactucopicrin (DHdLp) were quantified in stems of Mol and Gal landraces. Overall, the total STL content was significantly higher in Mol than Gal stems (84.9 ± 5.0 vs. 55.4 ± 3.0 mg kg^−1^ dry matter) as well as that of total lactucin-like forms (LcTOT, 66.2 ± 6.7 vs. 37.7 ± 4.5) independently of the growth site (Table 1, values in the “Both” line). The total lactucopicrin-like compounds showed comparable levels in both landraces (LpTOT, 18.6 ± 2.0 vs. 17.7 ± 2.5). The mean contents of DHLc and DHdLc were 2-fold higher in Mol than Gal (26.6 ± 2.8 vs. 11.1 ± 1.4 and 29.7 ± 2.8 vs. 12.2 ± 1.5, respectively). Vice versa, the Lc and dLc contents were slightly but significantly higher in Gal than Mol (6.4 ± 0.8 vs. 5.1 ± 0.8 and 8.0 ± 0.9 vs. 4.9 ± 2.5). Finally, the Lp amount was higher in Mol than Gal stems, whilst that of DHLp was comparable in the two genotypes. The environment change *per se* did not cause any significant variation of all compounds in both landraces (Table 1, values in the “A” and “L” lines). However, genotype-environment interactions were observed for the content of DHdLc, which decreased in Mol and was unvaried in Gal, and for Lc and Lp, which differed between Mol and Gal, only in one of the two sites (Lazio for Lc and Apulia for Lp). The principal component analysis (PCA, Figure 1) of STL data produced the two principal components PC1 and PC2 explaining, respectively, 80.67 and 19.30% of the total variance. The PCA fully separated Gal from Mol genotypes, respectively, located on the left and right side of PC1. Negative values of PC1 correlated with DHLp, Lc and dLc whilst the positive ones showed high correlation with the other compounds. Referring to PC2, Gal from Lazio (L) and Mol from Apulia (A) were in the upper quadrants, mainly positively correlated to the highest content of DHLp in Gal/L and of Lp and total STL in Mol/A (Table 1).

**Table 1:** **The sesquiterpene lactone (STL) content in stems of “Galatina” and “Molfettese” landraces**.

		**STLs content (mg kg^−1^ dry matter)^1^**								
**Genotype**	**Site^2^**	**Lc**	**DHLc**	**dLc**	**DHdLc**	**LcTOT**	**DHLp**	**Lp**	**LpTOT**	**TOTAL**
“Galatina”	A	5.9±0.9ab	10.3±1.5c	7.3±0.9	11.3±1.5c	34.7±4.6b	5.4±1.4	10.8±1.5b	16.2±2.9b	50.9±7.4b
	L	6.9±0.3a	12.0±0.8c	8.7±0.4	13.1±0.9c	40.6±2.2b	6.5±0.3	12.7±0.7ab	19.2±0.9a	59.9±3.1b
	Both	6.4±0.8	11.1±1.4	8.0±0.9	12.2±1.5	37.7±4.5	5.9±1.1	11.8±1.5	17.7±2.5	55.4±3.0
“Molfettese”	A	5.6±0.9ab	28.6±1.8a	5.3±3.8	31.9±2.1a	71.3±4.3a	5.3±0.7	14.7±1.0a	20.0±1.7a	91.3±5.8a
	L	4.7±0.4b	24.5±2.1b	4.4±0.6	27.5±1.0b	61.2±4.0a	4.5±0.6	12.8±0.9ab	17.3±1.4b	78.5±5.5a
	Both	5.1±0.8	26.6±2.8	4.9±2.5	29.7±2.8	66.2±6.7	4.9±0.7	13.7±1.3	18.6±2.0	84.9±5.0
**SIGNIFICANCE^3^**										
Genotype		^*^	^***^	^*^	^***^	^***^	ns	^*^	ns	^***^
Environment		ns	ns	ns	ns	ns	ns	ns	ns	ns
Gen. × Env.		^*^	^*^	ns	^**^	^*^	ns	^*^	^*^	^*^

1 *Lc, lactucin; DHLc, 11(S), 13-dihydrolactucin; dLc, 8-deoxylactucin; DHdLc, 11(S), 13-dihydro-8-deoxylactucin; Lp, lactucopicrin; DHLp, 11(s), 13-dihydrolactucopicrin; LcTOT, total lactucin-like STLs; LpTOT, total lactucopicrin-like STLs*.

2 *Cultivation site: A, Apulia; L, Lazio; Both, data from both growing sites were merged for a given genotype and mean values ± s.d. reported*.

3 *ns, non-significant*.

*, **, *** = *significant at P < 0.05, 0.01, and 0.001, respectively*.

*Genotype significance refers to both-site data; different letters within the same column indicate statistically significant differences in genotype X environment interactions*.

### Transcriptome assembly

*Cichorium intybus* RNA-seq libraries were prepared from the apical tips, stems, leaves and roots sampled at transplant and harvest stages of the Gal landrace (Table 2). The Illumina HiSeq2000 sequencing yielded approximately 164 million raw (100 bp) paired-end reads. The reads were processed to remove low-quality and adapter sequences, and ca. 97.7% of them (Table 2) were used as common dataset to follow two workflows, named “one-step” and “two-step” assembly strategies (Table 3 and Figure S1). In the former, the high-quality reads were assembled *de novo* by Trinity (Grabherr et al., 2011) into 80,303 contigs with a N50 and mean lengths of 1472 and 1,130.9 bp, respectively. The two-step strategy first included a template-based assembly and then a *de novo* one. Briefly, a non-redundant set of 26,085 unique sequences was generated by EGassembler pipeline (Masoudi-Nejad et al., 2006) using all the *C. intybus* public ESTs available at current date. Subsequently, the filtered reads were mapped on the unigenes EST set of 26,085 resulting into 11,153 read-supported EST fragments. These were used as input for iterative contig extension process using SeqMan Pro (DNAStar) which could raise the unigene mean length from ca. 760 to 1248 bp. Finally, the unmapped reads were retrieved by Bowtie2 (Langmead and Salzberg, 2012) and *de novo* assembled by Velvet/Oasis (Zerbino and Birney, 2008; Schulz et al., 2012) into 35,091 contigs. The output from both one- and two-step approaches were merged to obtain the final reference assembly consisting of 79,716 transcripts (N50 = 1545 bp and average contig length = 1230 bp) clustered into 58,872 isoform groups (unigenes). The unigenes set had a N50 and mean lengths of 1574 and 1220 bp, respectively (Table 3).

**Table 2:** ***C. intybus* RNA sequencing datasets**.

**Reference assembly**		
Genotype	“Galatina”	
Tissue	Apex, stem, leaf, root	
Stages	Transplant and harvest	
Site	Apulia	
Read types	2 × 100 bp	
Raw reads	164,768,038	
Cleaned reads	160,952,538	
Retained reads	97.7%	
RNA-RESEQUENCING^a^		
Genotype	“Galatina”	“Molfettese”
Tissue	Edible stem	Edible stem
Stages	Harvest	Harvest
**Growth site**	**Apulia**	
Replicates	3	3
Read types	1 × 50 bp	1 × 50 bp
Raw reads	19,469,829	18,953,475
Cleaned reads	19,424,671	18,864,361
Retained reads	99.8%	99.5%
**Growth site**	**Lazio**	
Replicates	3	3
Read types	1 × 50 bp	1 × 50 bp
Raw reads	30,296,567	23,953,335
Cleaned reads	30,176,328	23,872,902
Retained reads	99.6%	99.7%

a *Mean values for each group of triplicates*.

**Table 3:** **Features of assembled transcriptome**.

	**One-step**	**Two-step**	
	***de novo* (Trinity)**	**EST-based**	***de novo* (Velvet)**
Contig number	80,303	11,153	35,091
Overall alignment rate (%)	93.1	23.5	64.4
Transcriptome size (Mb)	90.9	13.9	42.7
Contig N50 length (bp)	1472	1452	1314
Contig mean length (bp)	1,130.9	1,247.5	1,216.3
**MERGED^a^**			
Contig number^b^	79,716		
Overall alignment rate (%)	95.7		
Transcriptome size (Mb)	98.1		
Contig N50 length (bp)	1545		
Contig mean length (bp)	1230		
Unigene number^c^	58,872		
Unigene N50 length (bp)	1574		
Unigenes mean length (bp)	1220		

a *One-step and two-step assembly strategies (described in the text) produced the merged transcriptome*.

b *Redundancy minimized by TGCIL/CAP3 pipeline*.

c *The longest transcripts were selected as representative for each cluster*.

### Annotation and function classification

**Figure 2:**
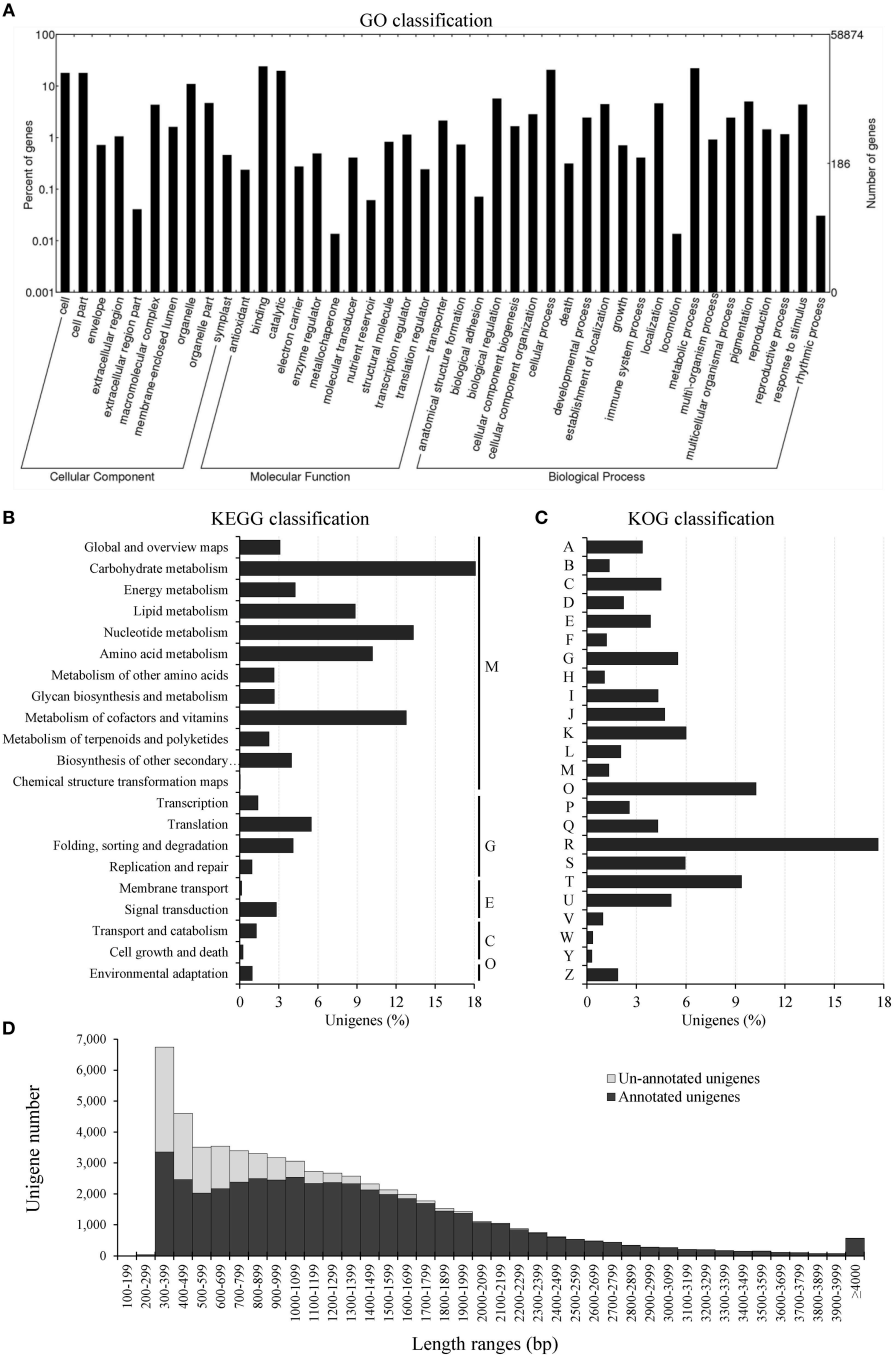
**Annotation of *Chicorium intybus* unigenes. (A)** GO classification. The GO terms were classified into three ontologies: biological process, cellular component, and molecular function. **(B)** KEGG classification. The histogram represents the unigene distribution into five major KEGG metabolic categories. M, metabolism; G, genetic information processing; E, environmental information processing; C, cellular processes; O, organismal systems. **(C)** Unigene functional classification into EuKaryotic Orthologous Groups (KOG). A, RNA processing and modification; B, Chromatin structure and dynamics; C, Energy production and conversion; D, Cell cycle control, cell division, chromosome partitioning; E, Amino acid transport and metabolism; F, Nucleotide transport and metabolism; G, Carbohydrate transport and metabolism; H, Coenzyme transport and metabolism; I, Lipid transport and metabolism; J, Translation, ribosomal structure and biogenesis; K, Transcription; L, Replication, recombination and repair; M, Cell wall/membrane/envelope biogenesis; N, Cell motility; O, Post-translational modification, protein turnover, chaperones; P, Inorganic ion transport and metabolism; Q, Secondary metabolites biosynthesis, transport and catabolism; R, General function prediction only; S, Function unknown; T, Signal transduction mechanisms; U, Intracellular trafficking, secretion, and vesicular transport; V, Defense mechanisms; Y, Nuclear structure; Z, Cytoskeleton. **(D)** Comparison of unigene length between annotated and non-annotated unigenes. The percentage of non-annotated transcripts (light gray bars) was high in the small-sized unigene category and progressively dropped along with the transcript length increase.

In order to widen the information on the newly assembled transcriptome, sequence similarity searches were performed against eight databases (see Material and Methods, and Table 4). The BlastX analyses showed that 38,931 unigenes (66.1%) had significant (*E*-value ≤ 10^−5^) matches in the Nr database, 38,978 (66.2%) in the TrEMBL, 36,281 (61.6%) in the Tair, and 26,233 (44.6%) in the SwissProt databases. GO, KEGG and KOG databases allowed functional classification of unigenes. An overview of the unigene annotations are in Table S3. Over 109,900 GO annotations were assigned to 23,501 unigenes (39.9%), and 7947 of them fell in the three ontology groups (Table 4, Figure 2A, and Figure S2). The “metabolic” and “cellular processes” were the most abundant categories (13,025 and 12,146 unigenes) within the “biological process;” “cell” and “cell part” top ranked (>10,500 unigenes) in the “cellular component,” while “binding” and “catalitic activity” included the highest unigene numbers (14,198 and 11,628) in the “molecular function” ontology. As for KEGG (Table 4 and Figure 2B), 7393 unigenes (12.6%) were mapped into 5 main categories and 130 metabolic maps. Most of the unigenes fell into the “metabolism” cluster (10,634; 82.7% of the mapped unigenes) followed by the genetic information processing, “environmental information processing,” “cellular processes” and “organismal systems” (11.9, 2.9, 1.5, and 0.9%, respectively). Within the “metabolisms,” those of “carbohydrate” (2416; 18.8%), “nucleotide” (1718; 13.4%) and “cofactors and vitamins” (1648; 12.8%) contained the highest number of unigenes. Based on the KOG database (Table 4 and Figure 2C), 11,650 unigenes (19.8%) belonged to 25 functional categories and the “general functional prediction only” (2309 unigenes; 17.6%), “post-translational modification, protein turnover, chaperones” (1341; 10.2%), and “signal transduction mechanisms” (1226; 9.4%), were the largest ones. InterProScan was used for structural annotation of the deduced products. The analysis used 15 databases and assigned 237,796 annotations to 31,516 unigenes (53.5%, in Table 4). The Interpro accessions (IPR, Table S4) were 20,642; 8935 putative proteins could be grouped into 2596 families, while 16,354 showed known domains, 1850 harbored repeats and 3308 hosted functional sites. Finally, the total unigenes of the *C. intybus* Gal transcriptome with at least one annotation signature were 45,572 (77.4%, in Table 4) and showed average length of 1372 bp; non-annotated unigenes were 13,302 (22.6%) and mostly of short size (ca. 700 bp; Figure 2D).

**Table 4:** **Number and percentage of annotated unigenes against public databases**.

**Database^a^**	**Unigenes n**.	**Unigenes %**
Nr	38,931	66.1
TrEMBL	38,978	66.2
Tair	36,281	61.6
InterPro	31,514	53.5
SwissProt	26,233	44.6
GO	23,501	39.9
KEGG	7393	12.6
KOG	11,650	19.8
Total	45,570	77.4

a *Nr, NCBI non-redundant database; TAIR, The Arabidopsis Information Resource; SwissProt is the manually annotated and reviewed section of the UniProt Knowledgebase (UniProtKB); TrEMBL, databases of UniProtKB automatically annotated and not reviewed; KOG, euKaryotic Ortholog Groups; Interpro, protein families database; GO, Gene Ontology; KEGG, Kyoto Encyclopedia of Genes and Genomes; Total, unigenes annotated in at least one database*.

### Digital gene expression and functional classification of differentially expressed genes

**Figure 3:**
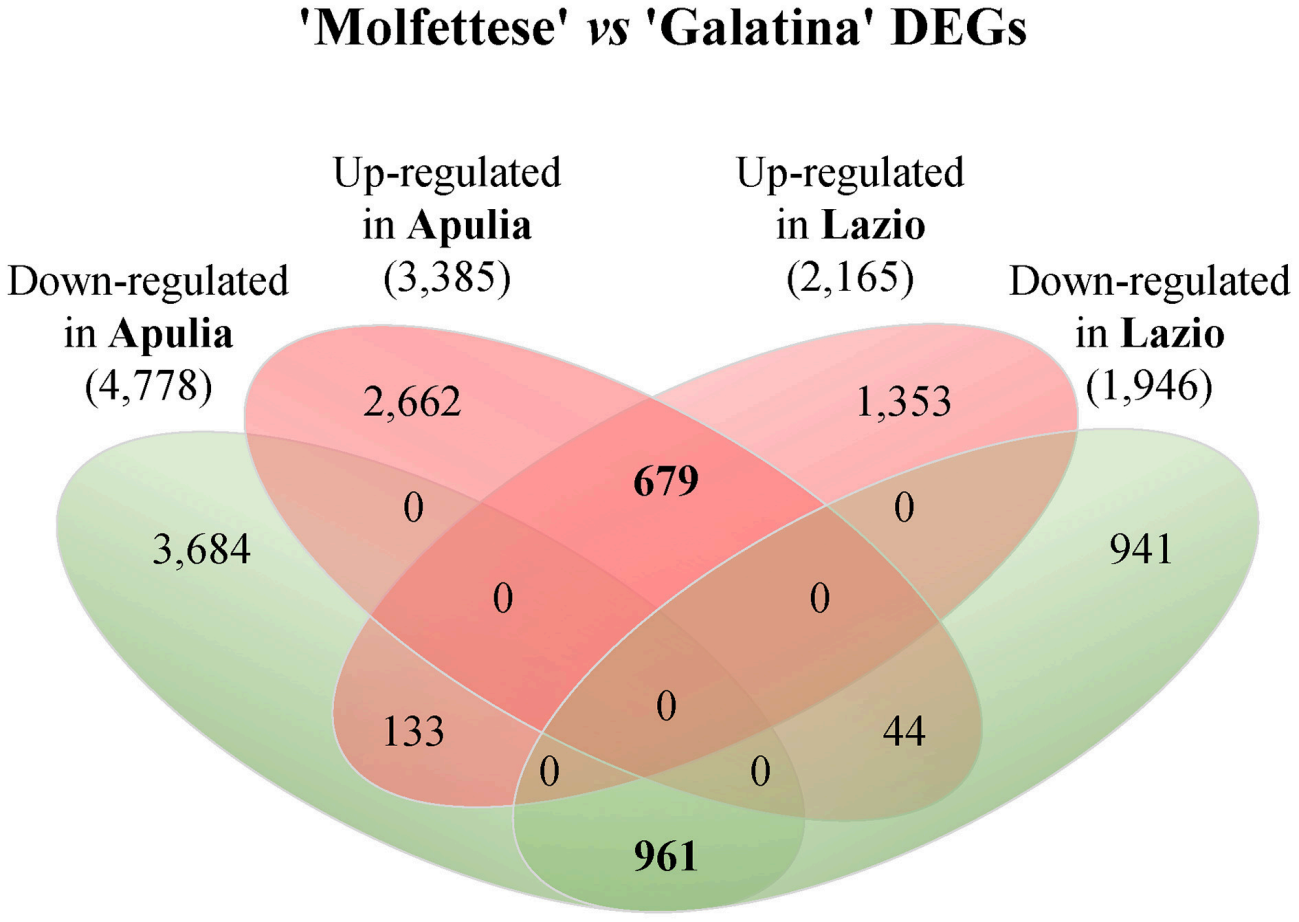
**Number of DEGs between Mol and Gal grown in Apulia and Lazio**. The numbers of up- (red bordered ellipsis) or down-regulated (green bordered ellipsis) DEGs in Mol with respect to Gal in both locations are reported. The number of DEGs with common or opposite expression trends are shown in the overlapping ellipsis areas. A total of 1640 (961 down-regulated plus 679 up-regulated) transcripts showed similar expression differences independently of the cultivation zone (bolded numbers).

Taken that Mol contained more STL than Gal, we first performed DGE profiling on the two genotypes within the same environment (inter-landrace comparison); subsequently, we selected the genes that maintained the relative expression pattern (independently of the area) for further characterization. 12,274 DEGs were identified (sum of up- and down- regulated unigenes bracketed in Figure 3); 6346 and 2294 DEGs were specific for Apulia and Lazio shires, respectively. Moreover, 1640 DEGs (961 down- plus 679 up-regulated unigenes, bolded in Figure 3) maintained the relative expression pattern independently from the cultivation zone, while 177 unigenes showed opposite trends from one site to the other. The transcriptome variation within a given genotype following cultivation site change is reported in Figure S3. KEGG (Table 5) and GO (Tables S5, S6) enrichment analyses were performed to functionally classify the 1640 DEGs. The former revealed that Mol up- and down-regulated genes were significantly over-represented in 9 and 4 pathways, respectively. Four up-regulated DEGs fell in the sesquiterpenoid and triterpenoid biosynthesis (Table 6).

**Table 5:** **KEGG pathway enrichment of differentially expressed genes in Mol vs. Gal**.

**Map**	**Description**	**Count^a^**	**Size^b^**	**FDR^c^**	**Rich factor^d^**
**UP-REGULATED**					
map00945	Stilbenoid, diarylheptanoid, and gingerol biosynthesis	4	18	6.83E-04	0.22
map01220	Degradation of aromatic compounds	1	6	2.40E-01	0.17
map00909	Sesquiterpenoid and triterpenoid biosynthesis	4	29	2.66E-03	0.14
map00941	Flavonoid biosynthesis	4	36	5.27E-03	0.11
map00940	Phenylpropanoid biosynthesis	19	258	2.01E-08	0.07
map00520	Amino sugar and nucleotide sugar metabolism	11	225	1.00E-03	0.05
map00360	Phenylalanine metabolism	12	248	6.83E-04	0.05
map00270	Cysteine and methionine metabolism	6	144	4.42E-02	0.04
map00500	Starch and sucrose metabolism	24	618	1.35E-05	0.04
**DOWN-REGULATED**					
map03440	Homologous recombination	3	28	2.88E-02	0.11
map00061	Fatty acid biosynthesis	5	68	1.77E-02	0.07
map03022	Basal transcription factors	4	58	2.88E-02	0.07
map00640	Propanoate metabolism	4	63	2.88E-02	0.06

a *DEGs number in the pathway; total up- and down-regulated genes were 97 and 72, respectively*.

b *Total number of genes referring to the specific pathway; the total gene number with KEGG annotation was 7393*.

c *False discovery rate, the table includes pathways with values ≤ 0.05*.

d *Ratio between the number of DEGs and genes annotated in a given pathway; higher rich factor values mean higher enrichment degree*.

**Table 6:** **Unigenes annotated in the sesquiterpenoid and triterpenoid pathway and DGE analysis**.

**EC number**	**Enzyme name**	**Unigenes**	**Size (bp)**	**DGE (RPKM)^a^**				**ER^b^**
				**Gal/A**	**Mol/A**	**Gal/L**	**Mol/L**	
1.14.13.120	Costunolide synthase	Ci_contig6325	2002	**25.2 ± 2.8**	**81.6 ± 2.3**	13.5±2.0	18.9±2.7	H
1.1.1.216	Farnesol dehydrogenase	Ci_contig486	4166	**15.4 ± 7.1**	**4.3 ± 4.1**	23.0 ± 3.5	25.7 ± 5.3	H
		Ci_contig52488	1358	15.5 ± 1.7	7.4 ± 1.1	10.4 ± 0.7	9.4 ± 1.8	H
1.1.1.314; 1.14.13.123	Germacrene A oxidase	Ci_contig7113	2486	**10.0 ± 0.3**	**23.8 ± 0.8**	**7.1 ± 0.6**	**15.0 ± 1.0**	H
1.14.13.121	Premnaspirodiene oxygenase	Ci_contig66434	1768	9.5 ± 7.5	7.0 ± 0.3	5.1 ± 0.6	4.9 ± 0.7	M
1.14.13.132	Squalene monooxygenase	Ci_contig52838	1825	46.9 ± 15.6	36.7 ± 3.1	48.3 ± 1.3	44.1 ± 6.7	H
		Ci_contig5712	2100	1.4 ± 0.3	1.6 ± 0.3	1.9 ± 0.0	1.5 ± 0.2	L
		Ci_contig34699	1322	34.3 ± 11.1	26.1 ± 2.4	44.7 ± 0.5	37.4 ± 5.9	H
2.5.1.21	Farnesyl-diphosphate farnesyltransferase	Ci_contig7389	1867	67.3 ± 5.8	82.1 ± 3.4	59 ± 3.3	59.4 ± 5.2	H
4.2.3.104; 4.2.3.57	α-humulene/β-caryophyllene synthase	Ci_contig56955	1382	0.1 ± 0.0	0.1 ± 0.1	0.1 ± 0.1	0.1 ± 0.1	L
4.2.3.23	Germacrene-A synthase	Ci_contig7229	3523	**6.1 ± 1.3**	**46.7 ± 5.4**	5.4 ± 0.3	8.4 ± 1.0	H
		Ci_contig14691	1850	0.1 ± 0.1	0.2 ± 0.1	0.1 ± 0.1	0.2 ± 0.1	L
		Ci_contig29105	1817	0.2 ± 0.1	0.1 ± 0.1	0.2 ± 0.1	0.0 ± 0.0	L
		Ci_contig62598	1827	**28.8 ± 1.5**	**75.0 ± 14.0**	**12.2 ± 2.4**	**27.0 ± 2.1**	H
		Ci_contig62597	1926	**9.7 ± 1.3**	**22.6 ± 2.7**	**3.7 ± 0.7**	**8.4 ± 0.2**	H
		Ci_contig73080	1937	0.0 ± 0.0	0.1 ± 0.0	0.2 ± 0.2	0.0 ± 0.1	N
4.2.3.39	Epi-cedrol synthase	Ci_contig41698	614	0.3 ± 0.1	0.1 ± 0.0	0.1 ± 0.0	0.1 ± 0.0	L
4.2.3.46	α-farnesene synthase	Ci_contig7791	1820	0.2 ± 0.1	0.0 ± 0.0	0.0 ± 0.0	0.1 ± 0.1	N
4.2.3.47	β-farnesene synthase	Ci_contig23130	677	0.1 ± 0.1	0.0 ± 0.0	0.1 ± 0.1	0.2 ± 0.1	N
		Ci_contig65366	1038	0.9 ± 0.0	0.8 ± 0.3	1.0 ± 0.1	1.4 ± 0.4	L
		Ci_contig65368	1550	0.5 ± 0.2	0.2 ± 0.1	0.6 ± 0.1	0.3 ± 0.1	L
		Ci_contig46748	321	0.2 ± 0.2	0.1 ± 0.1	0.0 ± 0.1	0.2 ± 0.2	L
4.2.3.48	(3S, 6E)-nerolidol synthase	Ci_contig10001	1595	**0.8 ± 0.4**	**3.6 ± 2.4**	0.4 ± 0.1	0.3 ± 0.1	L
4.2.3.57	β-caryophyllene synthase	Ci_contig10438	1552	**0.5 ± 0.1**	**1.9 ± 0.7**	0.5 ± 0.2	0.7 ± 0.2	L
4.2.3.75	(-)-germacrene D synthase	Ci_contig41699	366	0.2 ± 0.2	0.0 ± 0.0	0.0 ± 0.1	0.0 ± 0.0	N
5.4.99.38	Camelliol C synthase	Ci_contig77181	3492	1.2 ± 0.1	1.6 ± 0.3	1.6 ± 0.1	2.1 ± 0.3	L
5.4.99.39	β-amyrin synthase	Ci_contig3360	2648	**4.0 ± 0.3**	**23.1 ± 6.1**	**7.9 ± 1.9**	**16.2 ± 3.3**	H
		Ci_contig34609	1169	1.0 ± 0.4	1.3 ± 1.0	1.5 ± 0.3	1.6 ± 0.3	L
		Ci_contig70336	2468	0.5 ± 0.2	0.6 ± 0.3	**1.1 ± 0.3**	**4.2 ± 1.2**	L

a *DGE, digital gene expression; mean ± standard deviation was based on 3 replicates per growing site, per genotype; bolded values indicate significant differences (FDR ≤ 0.05; |log_2_ fold change| ≥ 1), gray shaded contigs indicate those genes with differential expression maintained in both planting sites*.

b *ER, expression range. H, high (RPKM > 8), M, moderate (RPKM 1–8), L low (RPKM 0.1–1) expression. N, below the expression threshold (RPKM 0–0.1)*.

### Dissections of putative genes related to sesquiterpenoid and triterpenoid pathway

**Figure 4:**
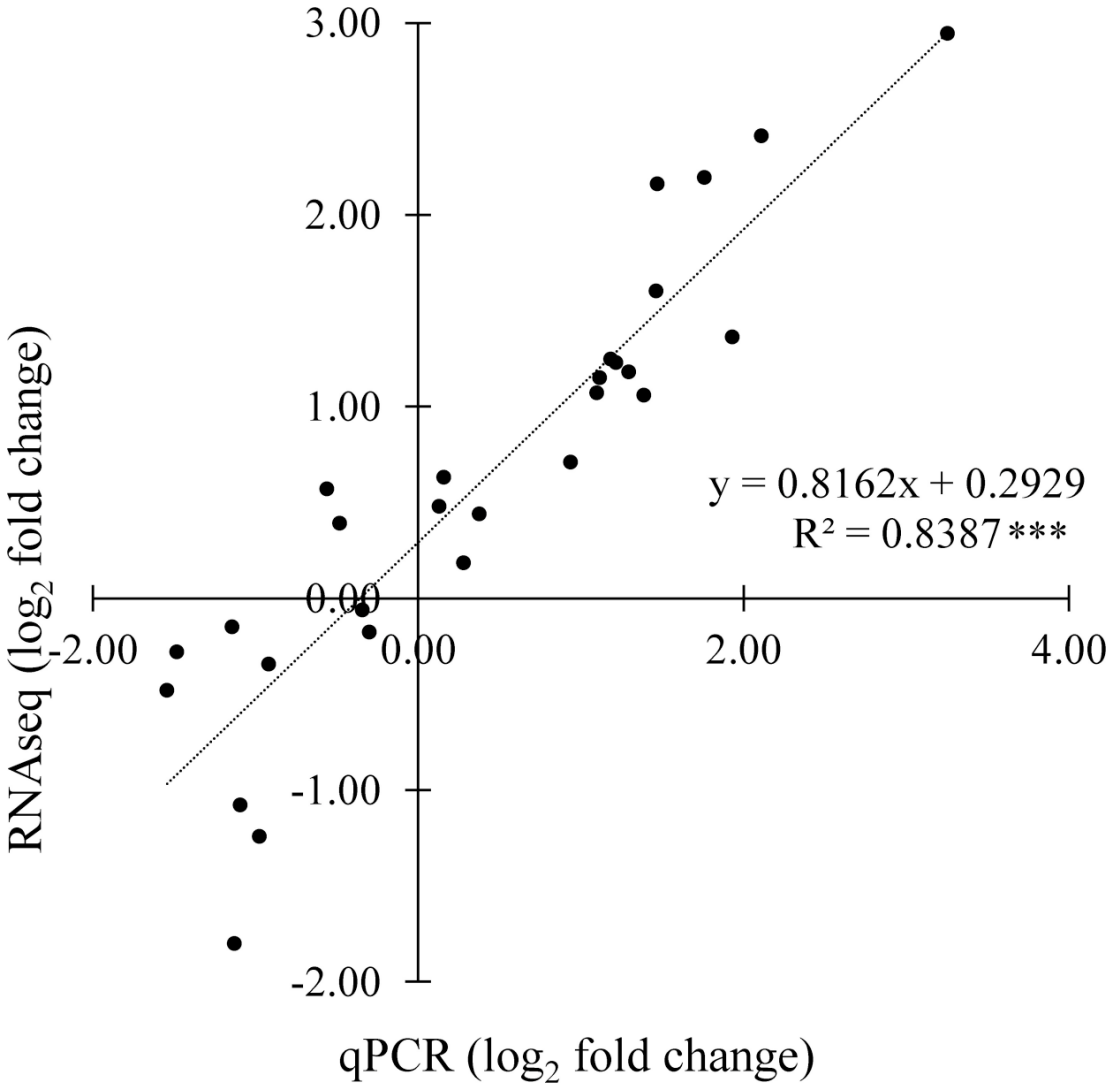
**Correlation of differential expression ratios (log_2_ fold change) obtained from RNAseq and quantitative PCR (qPCR) analyses**. Fourteen genes belonging to the sesquiterpenoid and triterpenoid pathway (Table S2) were arbitrarily chosen and their expression was monitored in Mol and Gal landraces in Apulia and Lazio areas. For a given unigene, RNAseq fold change refers to the ratios of RPKM values of Mol to Gal, whilst qPCR fold change is the relative expression of Mol normalized to those of Gal. *R*^2^, coefficient of determination; ***, significant correlation at *P* < 0.001.

**Figure 5:**
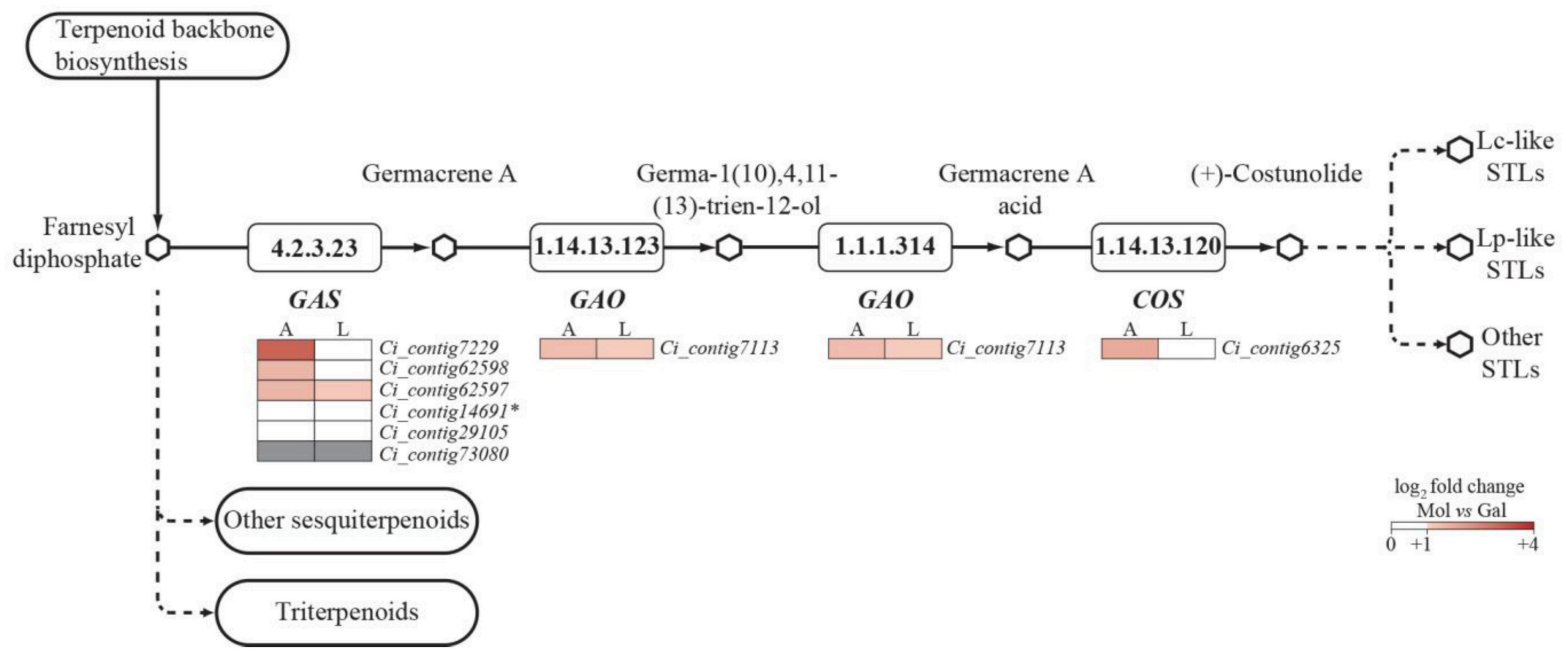
**Unigenes predicted to be involved in the Costunolide biosynthesis (a key precursor of Sesquiterpene lactones, STLs), in the Sesquiterpenoid and triterpenoid biosynthetic pathway**. Expression patterns of *GAS* (*Germacrene A synthase*), *GAO* (*Germacrene A oxidase*), and *COS* (*Costunolide synthase*) unigenes were monitored in Mol and Gal in both Apulia (A) and Lazio (L). Differential expression patterns (FDR ≤ 0.05; |log_2_ fold change| ≥ 1) between the two landraces are represented by color gradation. Gray boxes highlight unigenes not expressed in stems (average RPKM 0–0.1). The asterisk marks a partial sequence.

**Figure 6:**
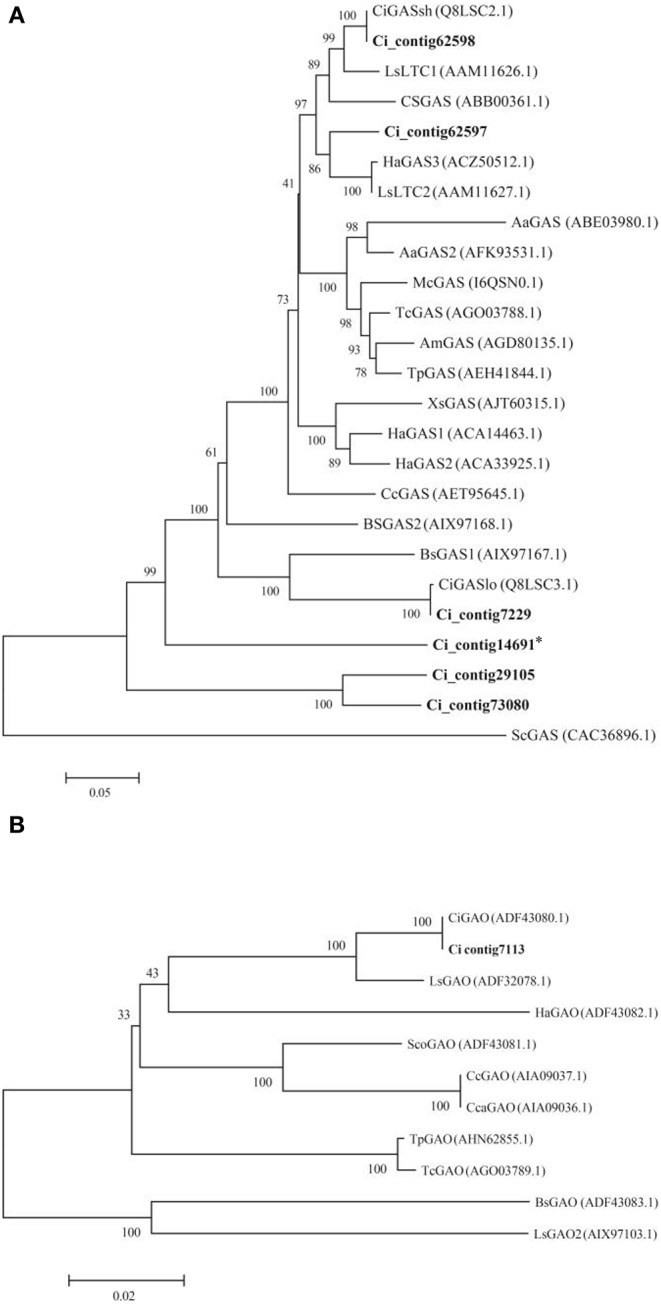
**Phylogenetic analysis of *C. intybus* GAS and GAO deduced proteins**. The trees were inferred with the neighbor-joining method. Bootstraps values (at the branching points) are given for major nodes and are based on 1000 replicates. The length of the lines indicates the relative distances between nodes. Putative proteins identified from *C. intybus* assembled transcriptome are in bold. GenBank accession numbers are shown in parentheses. **(A)** Phylogenetic tree of GAS proteins from *Asteraceae* species. Asterisk indicate a partial deduced product. **(B)** Phylogenetic tree of GAO proteins from *Asteraceae* species. Aa, *Artemisia annua*; Am, *Achillea millefolium*; Bs, *Barnadesia spinosa*; Cc, *Cynara cardunculus* var. *scolymus*; Cca, *Cynara cardunculus* var. *altilis*; Ci, *Cichorium intybus*; Cs, *Crepidiastrum sonchifolium*; Ha, *Helianthus annuus*; Ls, *Lactuca sativa*; Mc, *Matricaria chamomilla* var. *recutita*; Sc, *Solidago canadensis*; Sco, *Saussurea costus*; Tc, *Tanacetum cinerariifolium*; Tp, *Tanacetum parthenium*; Xs, *Xanthium strumarium*.

Sesquiterpene lactones (STL) precursors belong to the sesquiterpenoid and triterpenoid pathway (STP); therefore, the latter was further characterized by identifying 29 unigenes putatively encoding 16 distinct enzymes (Table 6 and Figure S4). Four unigenes were below the transcription threshold (RPKM 0–0.1), 13 showed a low expression (RPKM 0.1–3), 1 was moderately (RPKM 3–8) and 11 were highly (RPKM >8) expressed in the stems of both landraces (Table 6). DGE patterns were checked by qPCR assays (Figure S5) performed on 15 unigenes of the STP, and a significant positive correlation was found (Figure 4). Consequently, the DEG analysis (Table S7) identified 4 unigenes (gray-shaded in Table 6) which maintained a significantly higher expression in Mol than Gal regardless of growth sites. These genes were the *germacrene A synthase* (*GAS;* Ci_contig62597 and Ci_contig62598), *germacrene A oxidase* (*GAO;* Ci_contig7113) and β*-amyrin synthase* (*LUP4;* Ci_contig3360). GAS and GAO are two key enzymes in the synthesis of germacrene-type STLs and act consecutively in the upstream steps that generate the costunolide (Figure 5). Within the STL branch, 8 sequences were found in the Gal transcriptome, consisting of 6 *GAS* transcripts (Table S7), 1 *GAO* and 1 *costunolide synthase* (*COS*) mRNAs. The GAS, GAO, and COS proteins shared significant identities with several *Asteraceae* orthologues (Table S7); the sequence variability among the 6 GAS ranged from 53.1 to 88.6% (Figure S6). The *Asteraceae* GAS phylogenetic tree (Figure 6A) placed Ci_contig62598 and Ci_contig7229 products in the clades of *C. intybus* short and long variants, respectively. The Ci_contig62597 derived protein belonged to the *L. sativa* LTC2 group, while the Ci_contig29105 and Ci_contig73080 formed a clade *per se*, sharing 88.6% sequence identity (Figure S6). The *Asteraceae* GAO phylogenetic tree (Figure 6B) assigned the Ci_contig7113 product in the branch of *C. intybus* GAO with which shared 100% identity (not shown).

### Correlation analyses among GAS, GAO, putative transcription factors, and STLs

**Figure 7:**
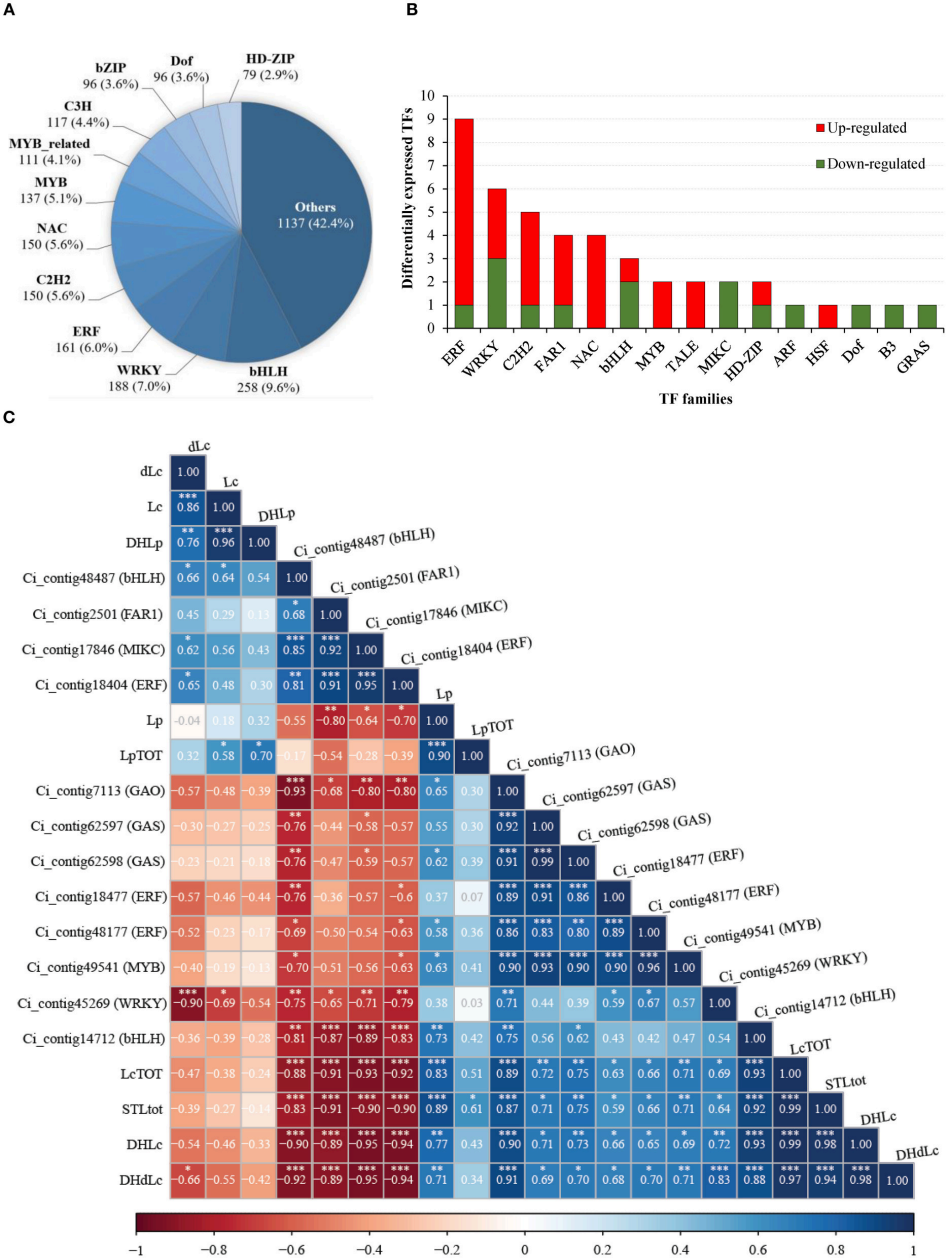
**Analysis of transcription factors (TFs) in *C. intybus* ‘Catalogna’ landraces. (A)** Categorization of predicted TFs into families. The numbers below each TF family (bold) indicate the number of unigenes within the group. Family relative abundance (percentage) with respect to the total predicted TFs is in parenthesis. **(B)** Analysis of differentially expressed (FDR ≤ 0.05; |log2FC| ≥ 1) TFs in Mol vs. Gal independently by the cultivation area. Red and green bars refer to up- and down-regulated TFs in Mol, respectively. **(C)** Correlogram representing Pearson's correlation coefficient (r) between content of STLs and gene expression abundances of both biosynthesis genes and putative TFs. Heat map is used to indicate the strength of correlation between the variables with ordering determined by hierarchical clustering. Red and blue indicate negative and positive correlations, respectively. Pearson's correlation coefficient values were reported into the colored squares. *, **, *** = significant at *P* ≤ 0.05, 0.01, and 0.001, respectively. Lc, lactucin; DHLc, 11(S), 13-dihydrolactucin; dLc, 8-deoxylactucin; DHdLc, 11(S), 13-dihydro-8-deoxylactucin; Lp, lactucopicrin; DHLp, 11(s), 13-dihydrolactucopicrin; STLtot, total STL content; LcTOT, total lactucin-like STL; LpTOT, total lactucopicrin-like STLs.

We approached the search for transcription factor genes (*TFs*) involved in the STL pathway. Globally, 2680 gene deduced products could be ascribed to 57 TF families according to PlantTFDB rules (Jin et al., 2014) and ca. 55% belonged to bHLH (258 proteins), WRKY (188), ERF (161), C2H2 (150), NAC (150), MYB (137), MYB-related (111), C3H (117), bZIP (96) and Dof (96) families (Figure 7A). Moreover, 46 TF genes maintained differential expressions in Mol vs. Gal independently of growth sites; most of DEGs fell into ERF (9), WRKY (6), C2H2 (5), FAR1 (4), NAC (4) and bHLH (4) groups (Figure 7B). Correlation analysis was carried out using the STL contents and the expression levels of *GAS* (Ci_contig62597, Ci_contig62598), *GAO* (Ci_contig7113) and the 46 *TFs* genes. A correlogram was built (Figure 7C) using 8 out of the 46 TF (see Table S2), which were selected by the criteria of transcript completeness and an absolute r value equal or greater than 0.75. Notably, the transcription of the biosynthesis genes *GAS* (Ci_contig62597, Ci_contig62598) and *GAO* (Ci_contig7113) showed very strong (*r* > 0.8) positive correlation between them and strong (0.7 ≤ *r* ≤ 0.8) correlation with total STL amounts (Figure 7C). As for *TF, GAS* and *GAO*, very strong positive correlations occurred between the expression of ERF (Ci_contig18477, Ci_contig48177) and *MYB* (Ci_contig49541) genes and both *GAS* and *GAO*. Conversely, strong (−0.8 ≤ *r* ≤ −0.7) and very strong (*r* < −0.8) negative correlations characterized the transcription of *bHLH* (Ci_contig48487) vs. that of *GAS* and *GAO*, while strong negative correlation of *MIKC* (Ci_contig17846) and *ERF* (Ci_contig18404) occurred just vs. *GAO*.

### Genotype differentiation by gene polymorphisms mining

Polymorphism identification from transcriptome sequences is useful to score gene functional variations in species without a sequenced genome. Consequently, SSR and SNP were mined and a specific focus was on the STL pathway genes. Overall, 11,672 putative SSRs were found in 9826 unigenes and 1525 of them contained more than one microsatellite (Table S8). Excluded mononucleotides, the di- and tri-nucleotide repeats were the most abundant (respectively 52.8 and 42.4% out of 6946 SSR); the AG/CT and ATC/ATG were the most frequent motifs (Table 7). The mapping of Mol reads vs. the Gal reference transcriptome scored 67,265 SNPs in 15,248 unigenes and ca. 68% were heterozygous (Table S9); the nucleotide substitutions (Figure S7) included 61.8% transition (C/T vs. A/G, 32.8 vs. 29%) and 38.2% transvertion events (A/T was the most frequent). The average SNP frequency occurred at 1/1068 bp, with a mean of 4.4 per unigene. Eleven out of the 29 unigenes of the sesquiterpenoid pathway (Table S9) bore SNPs between Gal and Mol landraces and 5 genes included homozygous SNPs. Referring to DEGs, β*-amyrin synthase* (*LUP4;* Ci_contig3360) showed 7 SNPs, while *GAS* (Ci_contig62598) contained one heterozygous SNP, which produced a synonym substitution (Tyrosin).

**Table 7:** **Summary of putative SSR identified in the ‘Galatina’ unigenes: sizes, frequencies and major types**.

**Unit repeat type**	**Number of repetitions**							**Total**	**Major type (%)**
	**5**	**6**	**7**	**8**	**9**	**10**	**>10**		
Di-nucleotide	0	1090	681	477	422	384	614	3668	AG/CT (62.9%)
Tri-nucleotide	1681	803	382	108	40	37	33	3084	ATC/ATG (24.3%)
Tetra-nucleotide	81	15	0	0	0	0	1	97	AAAT/ATTT (24.7%)
Penta-nucleotide	11	1	0	1	1	0	0	14	ACATG/ATGTC (21.4%)
Hexa-nucleotide	48	16	6	1	2	2	8	83	AAAAAG/CTTTTT (6.0%)

## Discussion

In this work, the combination of a *de novo* transcriptome assembly, transcript and metabolite profiling was used to achieve insights in the genetic pathway of sesquiterpenes and, more generally, valuable genetic tools using two stem-chicory “Catalogna” landraces.

As for total STL extraction procedure, cellulase treatment, and ultrasound assisted extraction were compared. Sonication can disrupt cell walls causing the release of cell contents (Toma et al., 2001) and it was effective as much as cellulose hydrolysis for yielding both free and bound fractions in chicory stems (Table S1). The metabolic characterization pointed that total STL content of stems was ca. 50-fold lower than that reported for “Catalogna” leaves (GEVES)^1^. Typically, STL have been either undetected or found in traces in stalks (Chou and Mullin, 1993; Douglas et al., 2004; Eljounaidi et al., 2015). Mol stems contained more total STL than those of Gal and differences were ascribed mostly to the genotype diversity and poorly to the environment changes; genotype-environment (GxE) interactions affected the contents of Lc, DHLc, DHdLc, and Lp variation (Table 1 and Table S1). Genotype effects were also observed in chicory forage, because cultivars with higher STL than others maintained the characteristics regardless of the growth sites (Foster et al., 2006). Chicory studies also report STL content variation with organ developmental stage, cultivation and geo-localisation that account for GxE effects (Peters et al., 1997; Foster et al., 2006; Ramirez et al., 2013; Chen et al., 2014). The higher content of DHLc, DHdLc and Lp in Mol vs. Gal may account for bitterness differences. The conversion of STL amounts into bitterness degrees (van Beek et al., 1990) indicates that Mol has higher scores than Gal (Table S10) and that the difference was mostly due to total Lc-like compounds. Simple gustative tests further assessed that Mol stems were more bitter than Gal ones (χ^2^ = 56.89; *p* < 0.001; Table S11). Consistently, the contents of Lc-like compounds showed positive correlation with bitterness in leaves of *C. intybus* sugarloaf, witloof and radicchio (Price et al., 1990; Peters and van Amerongen, 1998; Poli et al., 2002) and DHLc contents strongly correlated with root bitterness (Hance et al., 2007). Lp is one of the most bitter among chicory STLs (van Beek et al., 1990) and Lp-like molecules are strongly related to bitterness in other *Asteraceae* species, such as lettuce (*L. sativa*) and endive (*C. endive*) leaves (Seo et al., 2009; D'Antuono et al., 2016). Finally, a recent survey on endives proposes that bitterness depends on the balance between STL and phenolic contents and that the different compounds within these two categories can affect the trait both individually and in a complex manner (D'Antuono et al., 2016). Known that total phenolic contents are comparable in Gal and Mol stems (Renna et al., 2014), analytic studies are envisaged in addressing bitterness in these novel products.

Comparative analysis of Gal and Mol transcriptomes scored that ca. 2.8% unigenes (1640 out of 58,872 total unigenes) maintained differential expression pattern between the two genotypes in both Apulia and Lazio sites. This suggested that environment minimally or equally affected this gene pool, which may represent a source of transcriptional markers. Within this pool, four up-regulated DEGs (Table 5) fell in the sesquiterpenoid (2 *GAS* and 1 *GAO*) and triterpenoid (β*-amyrin synthase*) biosynthesis (Table S7). The diversity in the functional amino acid stretches among the 6 GAS deduced proteins (Figure S6) suggests that they may have diversified catalytic functions with substrate specificity. The two GAS enzymes encoded by the up-regulated DEGs are similar but not identical, and ascribed to the *C. intybus* short form (Figure 6 and Figure S6). Both *GAS* genes (Ci_contig62597 and Ci_contig62598) are 2-fold more expressed in Mol than Gal. Similarly, the *GAO* gene transcription is twice higher in Mol than Gal and the deduced protein corresponds to that of *C. intybus* available in public databases. Significant positive correlation occurred between *GAS* and *GAO* transcriptions and the contents of Lc-like (DHLc, DHdLc). These results corroborate the finding that terpene accumulation goes in parallel with the expressions of respective synthase genes (Nagegowda, 2010) and are consistent with the correlation of artichoke *GAS* and *GAO* transcription levels with the cyanopicrin-STL abundance (Eljounaidi et al., 2015). Regarding the *COS* gene (costunolide is a down-stream precursor of STL), it is worth noting that the expression was more abundant in Mol than Gal in Apulia; the trend was maintained in Lazio, but not at significant level (Table 6 and Figure S5).

The STL genetic pathway *per se* needs investigation in plants, namely, the biosynthesis genes downstream the *COS* (leading to Lc- and Lp-like compounds) have been unknown as well as the routes of catabolism and transport. Moreover, the knowledge on the TFs involved in sesqui- and triterpenoids biosynthesis is still fragmentary (Yamada and Sato, 2013). In this work, 46 *TF* genes conserved the differential expression between the two genotypes. Among these, some showed strong positive (*MYB, Ci_contig49541*) and negative (*bHLH, Ci_contig48487*) correlations with both the biosynthesis genes (*GAS* and *GAO*) and total STLs (three-way relationship). Given that MYB factors and bHLH members control sesquiterpenes synthesis in plants acting on terpene synthase genes (Hong et al., 2012; Reeves et al., 2012; Lu et al., 2013), it is proposed that the above-mentioned genes may represent TF that regulate the stem-chicory STL pathway. However, some TF showed significant strong correlations in two-way relationships. For instance, the *Ci_contig18404* (ERF) showed a very strong negative correlation (*r* < −0.8) with *GAO* and total STLs, but not with *GAS*. Similarly, the *Ci_contig45269* (WRKY) had a strong positive correlation with *GAO* but not with *GAS* or total STL. Consequently, these TFs might take part in STL metabolism of *C. intybus* through specific routes or indirectly, consistently with their role in regulating STL biosynthesis in other species (Yamada and Sato, 2013). Moreover, the future availability of *GAS* and *GAO* genomic sequences will allow prediction of motifs targeted by these candidate genes and pave the way for functional study experiments.

*Cichorium intybus* genome is esteemed ca. 1400 MB (De Simone et al., 1997), hence transcriptome sequencing was convenient to widen resources aimed to gene discovery, expression profiling, and diversity analysis and to marker production for breeding. There have been two Illumina-based transcriptomes of *C. intybus*, which derived from seedlings (Hodgins et al., 2014). The 'Galatina' reference transcriptome enriches the number of those within the leafy group—“Catalogna,” Witloof (Hodgins et al., 2014) and Radicchio—and widens the investigation spectrum because it is based on several mature and young vegetative tissues. In the absence of a genome sequence, it is recommended that the transcriptome construction of non-model species is achieved through the joining of reference-guided and *de novo* transcriptome assemblies (Ockendon et al., 2016) and/or the combinatorial use of different assemblers (Nakasugi et al., 2014). The assembly used in this work employed the one- and two-step strategies (Figure S1), which produced ca. 58,000 unigenes comparably to other *C. intybus* transcriptomes (Hodgins et al., 2014) but with nearly doubled length (1230 vs. 635–684 bp). Moreover, the Gal transcriptome contained 77.4% of annotated unigenes of average length of 1372 bp, consistently with features of other *Asteraceae* transcriptomes (Wang et al., 2013; Jung et al., 2014; Peng et al., 2014). The Gal transcriptome provided over 11,000 putative SSR markers; more than 15,000 unigenes differed between Gal and Mol for over 20,000 homozygous SNPs. The SSR and SNP validation by wide screening on different populations was beyond the scope of this work. However, the filtering criteria for polymorphism mining (Kumar et al., 2012) provide info to create SNP or SSR markers targeting alleles with variants in coding sequences. These marker types are useful to detect a causative mutation (Field and Wills, 1996) and are highly transferable across species (Varshney et al., 2005). The SNP frequency was 1/1068 bp suggesting that the two landraces are related. The polymorphisms events are expected to increase by screening a higher number of “Catalogna” landraces. As for SNPs in the STL pathway genes and the respective putative biological function, only a synonymous substitution was found in the *Ci_contig62598* (*GAS*) differentiating Gal and Mol unigenes. This implies that no protein mutation occurs that might explain the STL content differences. These latter are more likely due to a diversified gene expression regulation (e.g., residing in gene promoter regions). Nonetheless, the heterozygous SNP provides a tool for genetic mapping of this key gene in the stem-chicory populations.

Finally, the landrace comparative approach and data mining of transcriptome and metabolic variations were efficient to discover genes involved in STL pathway as a precious source to comprehend regulation of bitter taste in this vegetables and support plant breeding for product quality.

## Author contributions

DG was responsible for research costs and guided the work design and manuscript writing. GT carried out transcriptome assembly, differential gene expression analysis, polymorphism mining, qPCR validation, statistics and writing. GM contributed to transcriptome assembly and software usage. MG and MR performed statistical analysis on sesquiterpenes contents and gustative test. MG, GA and AS produced plant materials. GCT carried out the sesquiterpenes quantification. CN, GF, ED, MI performed sampling, phenotyping and nucleic acid extractions. All the authors reviewed, edited and approved the manuscript.

## Funding

This work was supported by a dedicated grant from the Italian Ministry of Economy and Finance to the National Research Council for the project “Innovazione e Sviluppo del Mezzogiorno–Conoscenze Integrate per Sostenibilità ed Innovazione del Made in Italy Agroalimentare” Law 191/2009 (www.mezzogiorno.cnr.it). CNR solutions n. 73 and n.74 of 29/3/2011 and pp. 1–26 of the annexed executive project.

### Conflict of interest statement

The authors declare that the research was conducted in the absence of any commercial or financial relationships that could be construed as a potential conflict of interest.

## Footnotes

^1^GEVES *Groupe d'Etudes et de Contrôle des Variétés et des Semences. The International Chicory Database, [Online]. European Cooperative Programme for Plant Genetic Resources*. Available online at: http://ecpgr.cgn.wur.nl/LVintro/chicory/download.htm [Accessed 2010].

## Abbreviations

COS: costunolide synthase
DEGs: differentially expressed genes
DGE: digital gene expression
DhdLc, 11(s): 13-dihydro-8-deoxylactucin
DHdLp, 11(s): 13-dihydrolactucopicrin
DHLc: 1,3-dihydrolactucin
dLc: 8-deoxylactucin
Gal: ‘Galatina’
GAO: germacrene A-oxidase
GAS: germacrene A-synthase
GO: gene ontology
Lc: lactucin
Lp: lactucopicrin
Mol: Molfettese'
PCA: principal component analysis
RPKM: reads per kilobases per million
SNPs: single nucleotide polymorphisms
SSR: simple sequence repeats
STL: sesquiterpene lactones
STP: sesquiterpenoid and triterpenoid pathway
TFs: transcription factors.
