# Corrigendum: Insights into the Sesquiterpenoid Pathway by Metabolic Profiling and *De novo* Transcriptome Assembly of Stem-Chicory (*Cichorium intybus* Cultigroup “Catalogna”)

**DOI:** 10.3389/fpls.2017.00478

**Published:** 2017-04-07

**Authors:** Giulio Testone, Giovanni Mele, Elisabetta Di Giacomo, Maria Gonnella, Massimiliano Renna, Gian Carlo Tenore, Chiara Nicolodi, Giovanna Frugis, Maria A. Iannelli, Giuseppe Arnesi, Alessandro Schiappa, Donato Giannino

**Affiliations:** ^1^Institute of Agricultural Biology and Biotechnology (IBBA), CNR - Monterotondo ScaloRome, Italy; ^2^Institute of Sciences of Food Production (ISPA), CNRBari, Italy; ^3^Department of Agricultural and Environmental Science (DiSAAT), University of BariBari, Italy; ^4^Department of Pharmacy, University of Naples Federico IINaples, Italy; ^5^Enza Zaden ItaliaTarquinia, Italy

**Keywords:** *Cichorium intybus*, stem-chicory landraces, transcriptome, sesquiterpene lactones, bitterness

There were these typing errors: “Mbp” (incorrect) instead of “cM” (correct) and “Bernardes” (incorrect) instead of “Bernardes” (correct).

In the Introduction, the sentence “The *C. intybus* species has a large (2n = 2x = 18; size 1405 Mbp) and complex genome (De Simone et al., 1997; Berardes et al., 2013).”

In the Discussion, the sentence (5th indent) “*Cichorium intybus* genome is esteemed ca.1400 MB (De Simone et al., 1997),”

were respectively corrected as follows:

“The *C. intybus* species has a large (2n = 2x = 18; size 1405 cM) and complex genome (De Simone et al., 1997; Bernardes et al., 2013).”

“*Cichorium intybus* genome is esteemed ca.1400 cM (De Simone et al., 1997).”

In the References section, the surname “Bernardes” was replaced with “Bernardes.”

The authors apologize for these errors and state that these do not change the scientific conclusions of the article in any way.

## Conflict of interest statement

The authors declare that the research was conducted in the absence of any commercial or financial relationships that could be construed as a potential conflict of interest.
