# Supplementary material for: Insights into the Sesquiterpenoid Pathway by Metabolic Profiling and De novo Transcriptome Assembly of Stem-Chicory (Cichorium intybus Cultigroup “Catalogna”)
Source: Front Plant Sci. 2016 Nov 8;7:1676. doi: 10.3389/fpls.2016.01676 (PMC5099503; doi:10.3389/fpls.2016.01676)
Supplement: Supplementary file 1 [file Table1.PDF]

Table S1

Comparison between cellulase treatment and ultrasound-assisted extraction (UAE) protocols for quantification of total (free and bound) sesquiterpene lactones (STL) in stems of 'Galatina' and 'Molfettese' landraces.

Genotype	Site[2]	Treatment[3]	STLs content (mg kg^{-1} dry matter)[1]								
			**Lc**	**DHLc**	**dLc**	**DHdLc**	**LcTOT**	**DHLp**	**Lp**	**LpTOT**	**TOTAL**
'Galatina'	A	Cellulase	5.5±0.4	9.8±1.1	6.9±0.6	10.9±1.2	33.1±6.5	5.2±1.6	10.3±1.2	15.5±2.3	48.7±9.0
		UAE	5.9±0.9	10.3±1.5	7.3±0.9	11.3±1.5	34.7±4.6	5.4±1.4	10.8±1.5	16.2±2.9	50.9±7.4
	L	Cellulase	6.5±0.8	11.5±1.1	8.3±0.8	12.5±1.2	38.9±3.8	6.3±0.8	12.3±1.3	18.5±1.3	57.5±5.1
		UAE	6.9±0.3	12.0±0.8	8.7±0.4	13.1±0.9	40.6±2.2	6.5±0.3	12.7±0.7	19.2±0.9	59.9±3.1
	Both	Cellulase	**6.0±0.4**	**10.7±0.7**	**7.6±0.5**	**11.7±0.8**	**36.0±3.4**	**5.7±0.8**	**11.3±0.8**	**17.0±1.2**	**53.1±4.7**
		UAE	**6.4±0.8**	**11.1±1.4**	**8.0±0.9**	**12.2±1.5**	**37.7±4.5**	**5.9±1.1**	**11.8±1.5**	**17.7±2.5**	**55.4±3.0**
'Molfettese'	A	Cellulase	5.4±1.0	27.8±2.5	5.2±2.3	30.8±3.2	69.2±4.0	5.2±1.1	14.5±1.5	19.7±2.0	88.9±5.3
		UAE	5.6±0.9	28.6±1.8	5.3±3.8	31.9±2.1	71.3±4.3	5.3±0.7	14.7±1.0	20.0±1.7	91.3±5.8
	L	Cellulase	4.6±0.5	23.8±3.1	4.3±0.8	27.0±2.3	59.6±5.1	4.5±1.0	12.6±1.6	17.0±2.5	76.5±5.0
		UAE	4.7±0.4	24.5±2.1	4.4±0.6	27.5±1.0	61.2±4.0	4.5±0.6	12.8±0.9	17.3±1.4	78.5±5.5
	Both	Cellulase	**5.0±0.5**	**25.8±2.0**	**4.7±1.1**	**28.9±1.9**	**64.4±3.0**	**4.9±0.72**	**13.5±1.2**	**18.3±1.6**	**82.7±3.4**
		UAE	**5.1±0.8**	**26.6±2.8**	**4.9±2.5**	**29.7±2.8**	**66.2±6.7**	**4.9±0.7**	**13.7±1.3**	**18.6±2.0**	**84.9±5.0**
**Significance**			ns	ns	ns	ns	ns	ns	ns	ns	ns

1, Lc, lactucin; DHLc, 11(S),13-dihydrolactucin; dLc, 8-deoxylactucin; DHdLc, 11(S),13-dihydro-8-deoxylactucin; Lp, lactucopicrin; DHLp, 11(s),13-dihydrolactucopicrin; LcTOT, total lactucin-like STLs; LpTOT, total lactucopicrin-like STLs.

2, Cultivation site: A, Apulia; L, Lazio; Both, data from both growing sites were merged for a given genotype and mean values ± s.d. reported.

3, In order to release glycoside (bound) STL, the crude extracts were resuspended in methanol and subject to cellulase treatment or to ultrasound-assisted extraction (UAE). ns, non-significant.
