# Supplementary material for: Insights into the Sesquiterpenoid Pathway by Metabolic Profiling and De novo Transcriptome Assembly of Stem-Chicory (Cichorium intybus Cultigroup “Catalogna”)
Source: Front Plant Sci. 2016 Nov 8;7:1676. doi: 10.3389/fpls.2016.01676 (PMC5099503; doi:10.3389/fpls.2016.01676)
Supplement: Supplementary file 2 [file Table2.PDF]

Table S2. List of primer used in qPCR.

Unigenes	Annotations	Symbol	Forward/Reverse primers (5' to 3')
**Ci_contig6325**	*Costunolide synthase*	COS	CATCATGCACCTACAGCTCGG<br>CATCTGCCTCCAGTATTCGCC
**Ci_contig66434**	*Premnaspirodiene oxygenase*	CYP71D55	CCATTTCGTCCACTGCAAGGC<br>TTCCGATACAGTACAAGATCACTCCCA
**Ci_contig41698**	*Epi-cedrol synthase*	ECS	AGAGACAATCGGAGGGAAGGG<br>TGACTTCACCTGGCATCCTC
**Ci_contig52488**	*Farnesol dehydrogenase*	FLDH	TGCTGGGAATGTAGTAGCCC<br>TGTGTTGGTGATTGTGGCAG
**Ci_contig7113**	*Germacrene-A oxidase*	GAO	TAGCACCTCAAGCAAAGCCAACG<br>CCGTTCAATGCTTGCCTTAGTTCAG
**Ci_contig7229**	*Germacrene-A synthase*	GAS	CAATTCCAACAATCCGTCCCT<br>ACGCTCTTCCGTCCTTTGAGTA
**Ci_contig62597**	*Germacrene-A synthase*	GAS	CCATCCAAGCGAAGACCAATACC<br>TTGCTCTAGCATATCCTTCCAACCC
**Ci_contig62598**	*Germacrene-A synthase*	GAS	ACCTTCAAGAAATGGCAGCAGT<br>AGCCTAACATACCCTTCACATCG
**Ci_contig3360**	*β-amyrin synthase*	LUP4	CACAAACAACCGCCACAAGCA<br>CATGATGGGATGGCGGTGA
**Ci_contig10001**	*(3S,6E)-nerolidol synthase*	NES	GCGTCTGTGGGATGATTTGGG<br>GTTGGTGGCATTCGTGAAGGTTAC
**Ci_contig10438**	*β -caryophyllene synthase*	QHS	GTCGTTCAAATGGGCTCTTACC<br>GCGTCCTTTCTTTCTCTTCCTG
**Ci_contig5712**	*Squalene monooxygenase*	SQLE	TGCTACCTTTACCCTCACCTAAC<br>GAACATTTGCCTCACTCCTTCC
**Ci_contig65366**	*β -farnesene synthase*	TPS10	GGCGATCACTGGATGGATGACT<br>CACCATTTGCTGATTTGGCTGA
**Ci_contig56955**	*α-humulene/ β-caryophyllene synthase*	TPS21	TGCACAGCAAGTGAAACACG<br>TCTCATCTCCTCAACGGTAGC
