# Supplementary material for: Insights into the Sesquiterpenoid Pathway by Metabolic Profiling and De novo Transcriptome Assembly of Stem-Chicory (Cichorium intybus Cultigroup “Catalogna”)
Source: Front Plant Sci. 2016 Nov 8;7:1676. doi: 10.3389/fpls.2016.01676 (PMC5099503; doi:10.3389/fpls.2016.01676)
Supplement: Supplementary file 8 [file Table8.PDF]

Table S8. SSR mining statistics.

Item	Number
Total number of sequences examined	58,874
Total size of examined sequences (bp)	71,830,461
Total number of identified SSRs	11,672
Number of SSR containing sequences	9,826
Number of sequences containing more than 1 SSR	1,525
Number of SSRs present in compound formation	543
