# Supplementary material for: Insights into the Sesquiterpenoid Pathway by Metabolic Profiling and De novo Transcriptome Assembly of Stem-Chicory (Cichorium intybus Cultigroup “Catalogna”)
Source: Front Plant Sci. 2016 Nov 8;7:1676. doi: 10.3389/fpls.2016.01676 (PMC5099503; doi:10.3389/fpls.2016.01676)
Supplement: Supplementary file 10 [file Table10.PDF]

Table S10

The deduced bitterness score of sesquiterpene lactones (STL) in stems of 'Galatina' and 'Molfettese' landraces.

		STL bitterness score[1]								
**Genotype**	**Site**[2]	**Lc**	**DHLc**	**dLc**	**DHdLc**	**LcTOT**	**DHLp**	**Lp**	**LpTOT**	**TOTAL**
'Galatina'	A	3.4±0.5 ab	7.4±1.1 c	6.6±0.7	10.3±1.4 c	27.7±3.7 b	27.0±7.0	21.6±3.0 b	48.6±10.0 b	76.3±13.7 c
	L	4.1±0.2 a	8.6±0.5 c	7.9±0.4	11.91±0.8 c	32.4±1.8 b	32.5±1.3	25.5±1.3 ab	58.9±2.4 a	90.4±4.1 bc
'Molfettese'	A	3.2±0.5 b	20.4±1.3 a	4.8±3.5	28.7±1.9 a	57.2±7.2 a	26.5 ±3.5	29.2±2.0 a	55.7±5.5 a	112.9±12.7 a
	L	2.8±0.3 b	17.5±1.5 b	4.0 ±0.5	25.0±0.9 b	49.3±3.1 a	22.7±2.8	25.5±1.8 ab	48.2±4.6 b	97.5±7.7ab
**Significance**[3]										
Genotype		*	***	*	***	***	ns	*	ns	**
Environment		ns	ns	ns	ns	ns	ns	ns	ns	ns
Gen. x Env.		*	*	ns	**	*	ns	*	*	*

1, Bitterness scores were calculated by dividing the concentration of each STL by its bitter threshold in water as measured by Van Beek et al. (1990). Scores are reported as mean values ± standard deviation.Lc, lactucin; DHLc, 11(S),13-dihydrolactucin; dLc, 8-deoxylactucin; DHdLc, 11(S),13-dihydro-8-deoxylactucin; Lp, lactucopicrin; DHLp, 11(s),13-dihydrolactucopicrin; LcTOT, total lactucin-like STLs; LpTOT, total lactucopicrin-like STLs.

2, Cultivation site: A, Apulia; L, Lazio;

3, ns, non-significant.*, **, *** = significant at P < 0.05, 0.01 and 0.001, respectively. Genotype significance refers to both-site data; different letters within the same column indicate statistically significant differences in genotype X environment interactions.
