# Supplementary material for: Insights into the Sesquiterpenoid Pathway by Metabolic Profiling and De novo Transcriptome Assembly of Stem-Chicory (Cichorium intybus Cultigroup “Catalogna”)
Source: Front Plant Sci. 2016 Nov 8;7:1676. doi: 10.3389/fpls.2016.01676 (PMC5099503; doi:10.3389/fpls.2016.01676)
Supplement: Supplementary file 11 [file Table11.PDF]

Table S11

Bitterness panel test of 'Galatina' and 'Molfettese' stems.

	More Bitter Taste[1]							
	**Expected**			**Observed**			$(O-E)^2/E$	$(O-E)^2/E$
	yes	no	total	yes	no	total	yes	no
'Galatina'	18	18	36	2	34	36	14.22	14.22
'Molfettese'	18	18	36	34	2	36	14.22	14.22
$\chi 2$	56.88							
p-value[2]	<0.001							

1, 36 assayers answered the question "Which of the two samples is more bitter?"; each assayer tasted 3 stems taken from a bulk containing samples from Apulia and Lazio in equal amounts.

2, one degree of freedom
