# Supplementary material for: Insights into the Sesquiterpenoid Pathway by Metabolic Profiling and De novo Transcriptome Assembly of Stem-Chicory (Cichorium intybus Cultigroup “Catalogna”)
Source: Front Plant Sci. 2016 Nov 8;7:1676. doi: 10.3389/fpls.2016.01676 (PMC5099503; doi:10.3389/fpls.2016.01676)
Supplement: Supplementary file 12 [file Presentation1.pptx]

## Slide 1
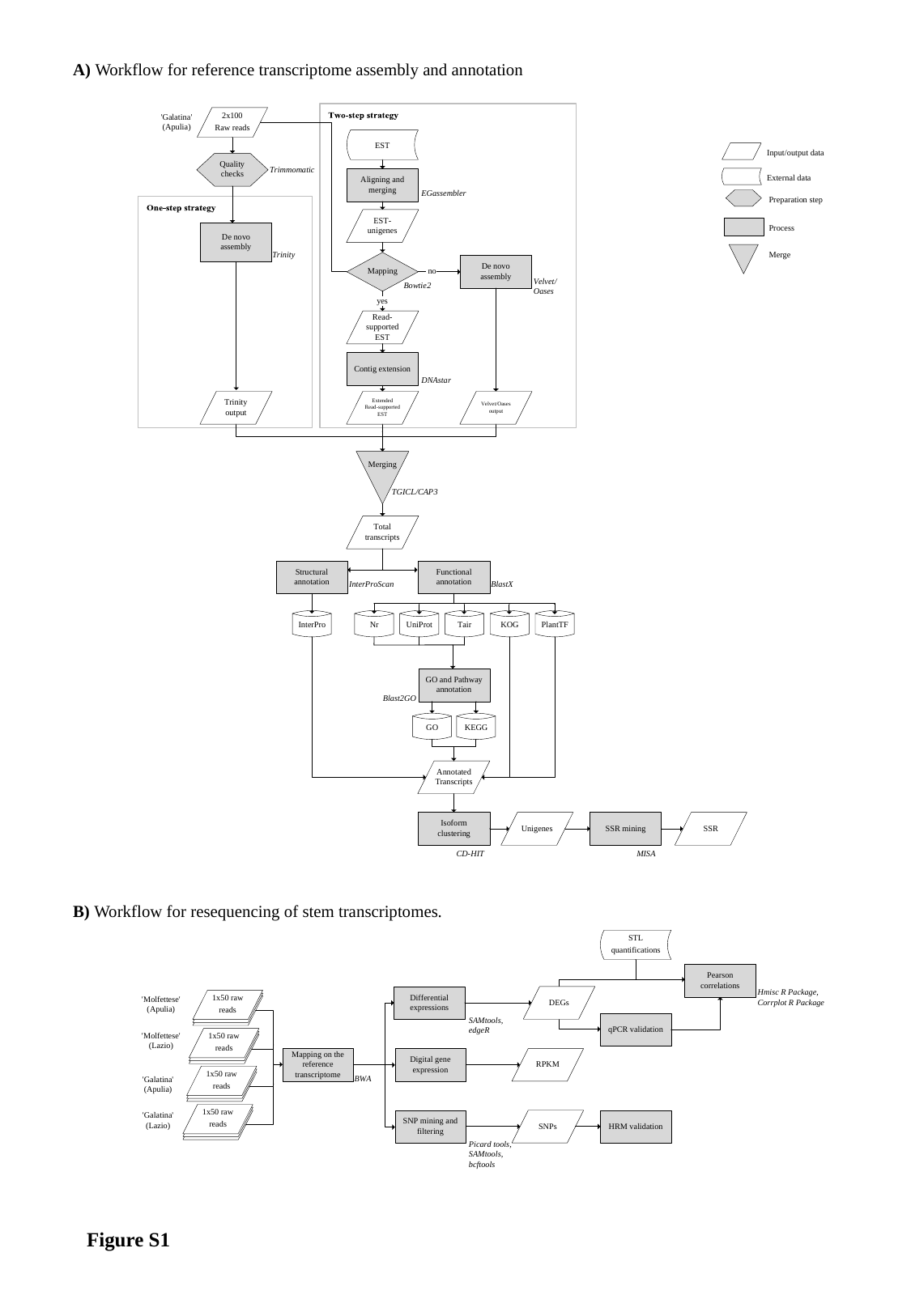

A) Workflow for reference transcriptome assembly and annotation
B) Workflow for resequencing of stem transcriptomes.
Figure S1

## Slide 2
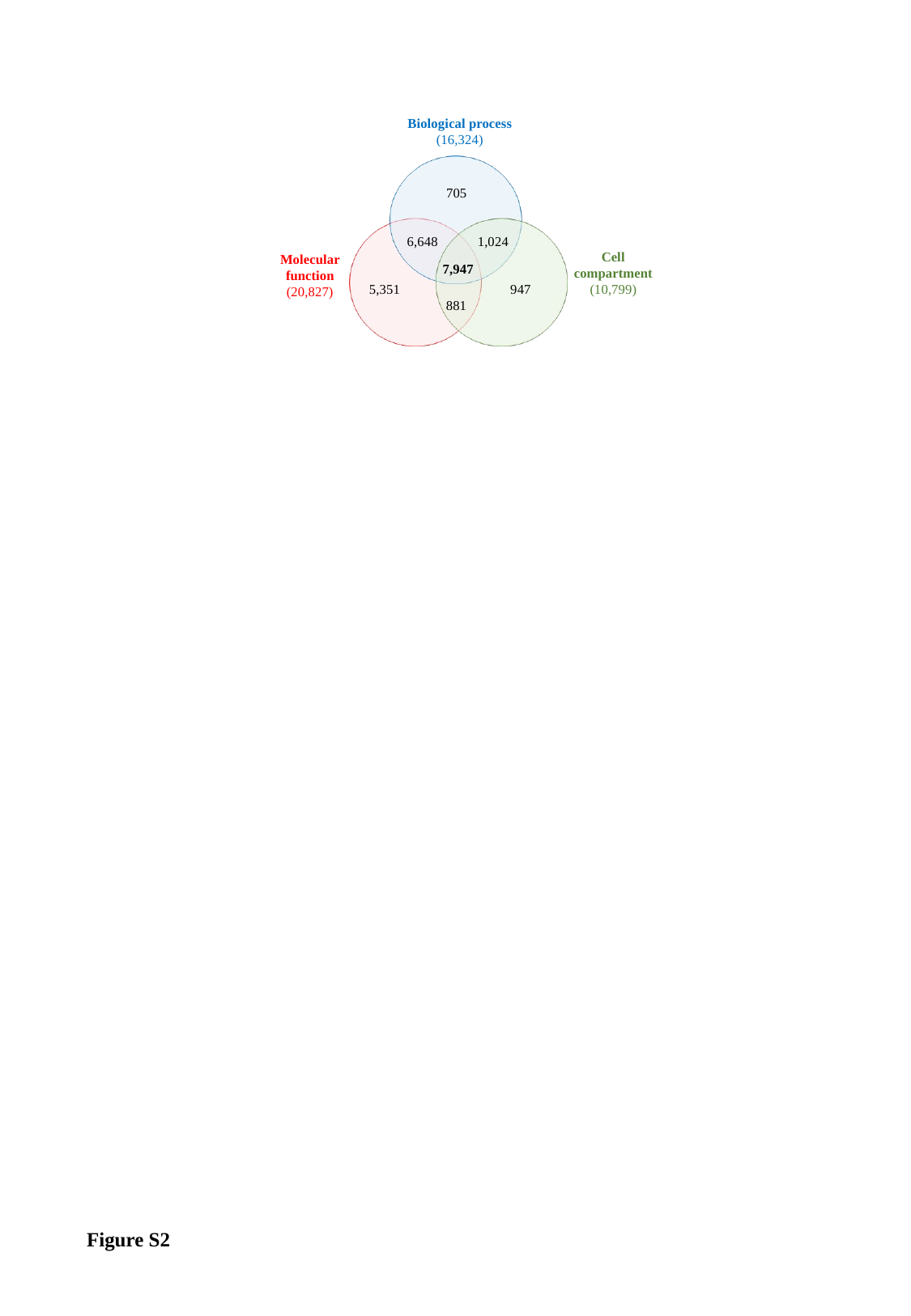

Biological process
(16,324)
705
6,648
1,024
Cell compartment
(10,799)
Molecular function
(20,827)
7,947
5,351
947
881
Figure S2
Figure S1

## Slide 3
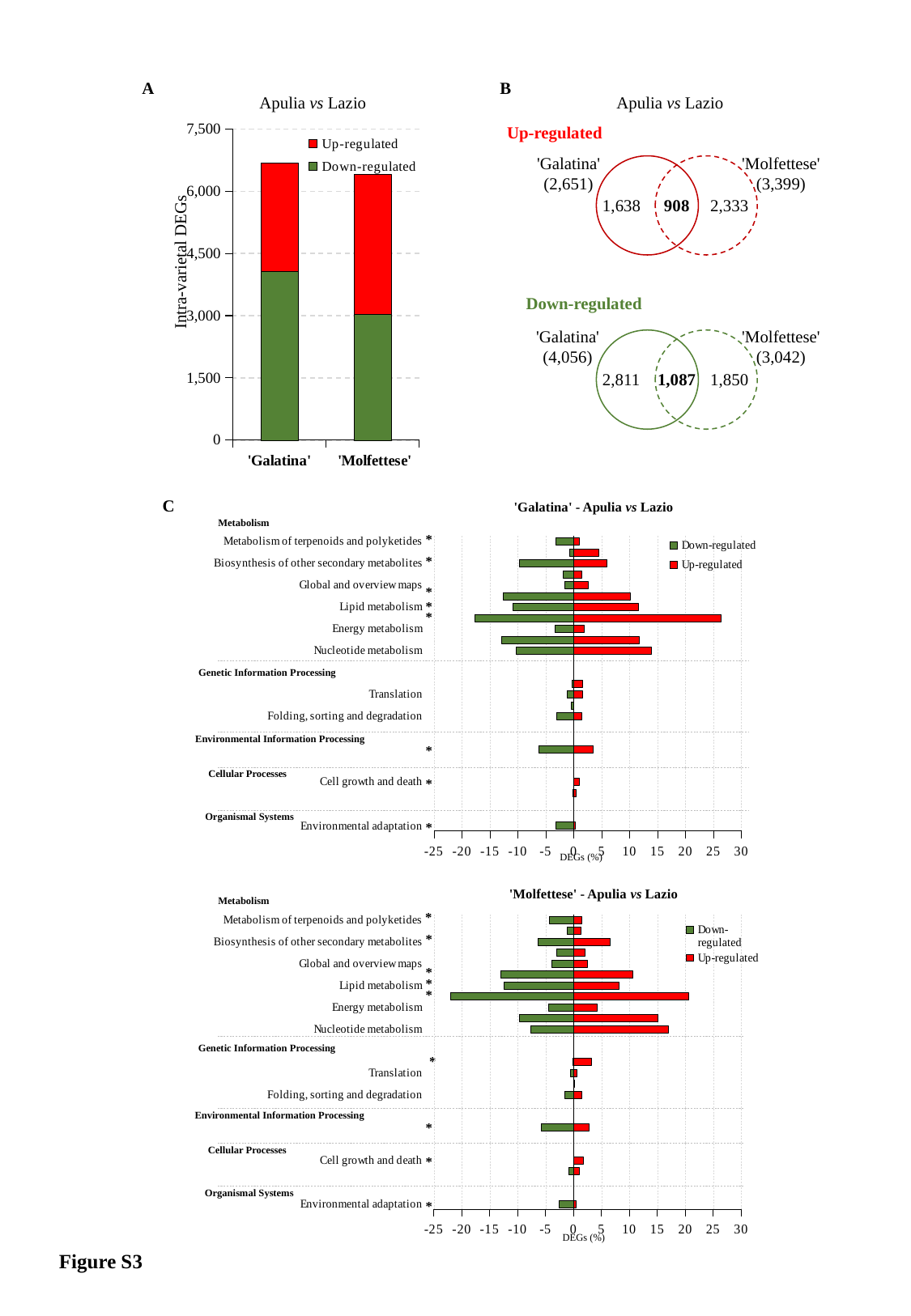

A
B
Apulia vs Lazio
Apulia vs Lazio
### Chart
| Category | | |
|---|---|---|
| 'Galatina' | 4061.0 | 2631.0 |
| 'Molfettese' | 3041.0 | 3377.0 |Up-regulated
'Galatina'
(2,651)
'Molfettese' (3,399)
1,638
908
2,333
Intra-varietal DEGs
Down-regulated
'Galatina'
(4,056)
'Molfettese' (3,042)
2,811
1,087
1,850
C
'Galatina' - Apulia vs Lazio
Metabolism
### Chart
| Category | Down-regulated | Up-regulated |
|---|---|---|
| Environmental adaptation | -3.202846975088968 | 0.2785515320334262 |
| | 0.0 | 0.0 |
| | 0.0 | 0.0 |
| Transport and catabolism | -0.1779359430604982 | 0.4178272980501393 |
| Cell growth and death | 0.0 | 0.9749303621169917 |
| | 0.0 | 0.0 |
| | 0.0 | 0.0 |
| Signal transduction | -6.227758007117438 | 3.4818941504178276 |
| | 0.0 | 0.0 |
| | 0.0 | 0.0 |
| Folding, sorting and degradation | -3.0249110320284696 | 1.392757660167131 |
| Transcription | -0.5338078291814947 | 0.0 |
| Translation | -1.2455516014234875 | 1.532033426183844 |
| Replication and repair | -0.3558718861209964 | 1.532033426183844 |
| | 0.0 | 0.0 |
| | 0.0 | 0.0 |
| Nucleotide metabolism | -10.320284697508896 | 13.788300835654596 |
| Metabolism of cofactors and vitamins | -12.98932384341637 | 11.699164345403899 |
| Energy metabolism | -3.3807829181494666 | 1.8105849582172702 |
| Carbohydrate metabolism | -17.793594306049823 | 26.323119777158777 |
| Lipid metabolism | -10.85409252669039 | 11.559888579387186 |
| Amino acid metabolism | -12.633451957295375 | 10.027855153203342 |
| Global and overview maps | -1.601423487544484 | 2.5069637883008355 |
| Metabolism of other amino acids | -1.9572953736654803 | 1.392757660167131 |
| Biosynthesis of other secondary metabolites | -9.786476868327403 | 5.8495821727019495 |
| Glycan biosynthesis and metabolism | -0.7117437722419928 | 4.456824512534819 |
| Metabolism of terpenoids and polyketides | -3.202846975088968 | 0.9749303621169917 |*
*
*
*
*
*
*
*
Genetic Information Processing
Environmental Information Processing
Cellular Processes
Organismal Systems
DEGs (%)
'Molfettese' - Apulia vs Lazio
Metabolism
Genetic Information Processing
Environmental Information Processing
Cellular Processes
Organismal Systems
### Chart
| Category | Down-regulated | Up-regulated |
|---|---|---|
| Environmental adaptation | -2.6775320139697323 | 0.375 |
| | 0.0 | 0.0 |
| | 0.0 | 0.0 |
| Transport and catabolism | -0.9313154831199069 | 1.0 |
| Cell growth and death | 0.0 | 1.7500000000000002 |
| | 0.0 | 0.0 |
| | 0.0 | 0.0 |
| Signal transduction | -5.820721769499418 | 2.75 |
| | 0.0 | 0.0 |
| | 0.0 | 0.0 |
| Folding, sorting and degradation | -1.629802095459837 | 1.375 |
| Transcription | 0.0 | 0.125 |
| Translation | -0.5820721769499418 | 0.5 |
| Replication and repair | -0.11641443538998836 | 3.125 |
| | 0.0 | 0.0 |
| | 0.0 | 0.0 |
| Nucleotide metabolism | -7.683352735739232 | 16.875 |
| Metabolism of cofactors and vitamins | -9.662398137369035 | 15.0 |
| Energy metabolism | -4.540162980209546 | 4.125 |
| Carbohydrate metabolism | -22.0023282887078 | 20.625 |
| Lipid metabolism | -12.456344586728754 | 8.125 |
| Amino acid metabolism | -13.038416763678695 | 10.625 |
| Global and overview maps | -3.958090803259604 | 2.5 |
| Metabolism of other amino acids | -3.026775320139697 | 2.0 |
| Biosynthesis of other secondary metabolites | -6.402793946449361 | 6.5 |
| Glycan biosynthesis and metabolism | -1.1641443538998837 | 1.25 |
| Metabolism of terpenoids and polyketides | -4.307334109429569 | 1.375 |*
*
*
*
*
*
*
*
*
DEGs (%)
Figure S3

## Slide 4
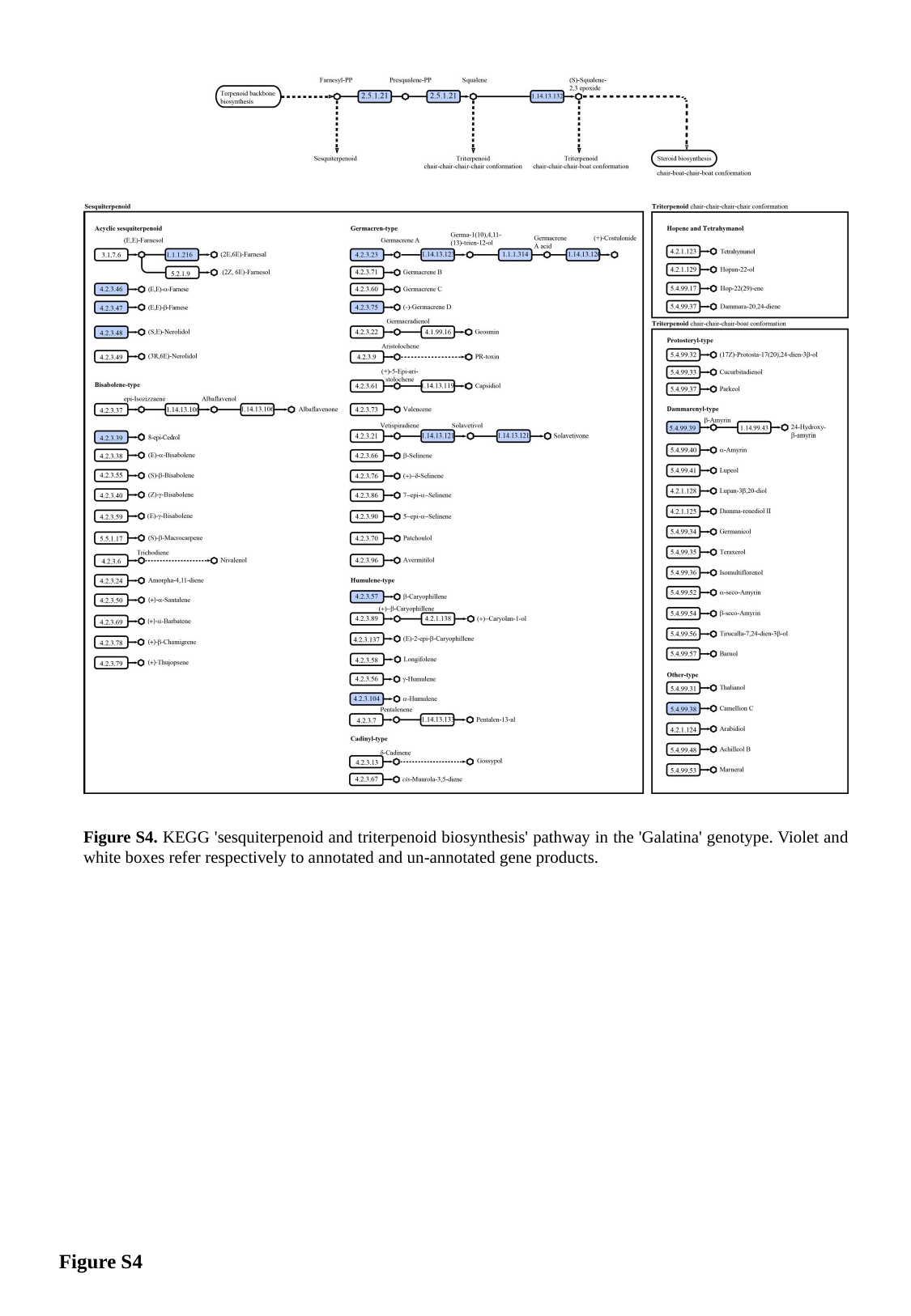

Figure S4. KEGG 'sesquiterpenoid and triterpenoid biosynthesis' pathway in the 'Galatina' genotype. Violet and white boxes refer respectively to annotated and un-annotated gene products.
Figure S4

## Slide 5
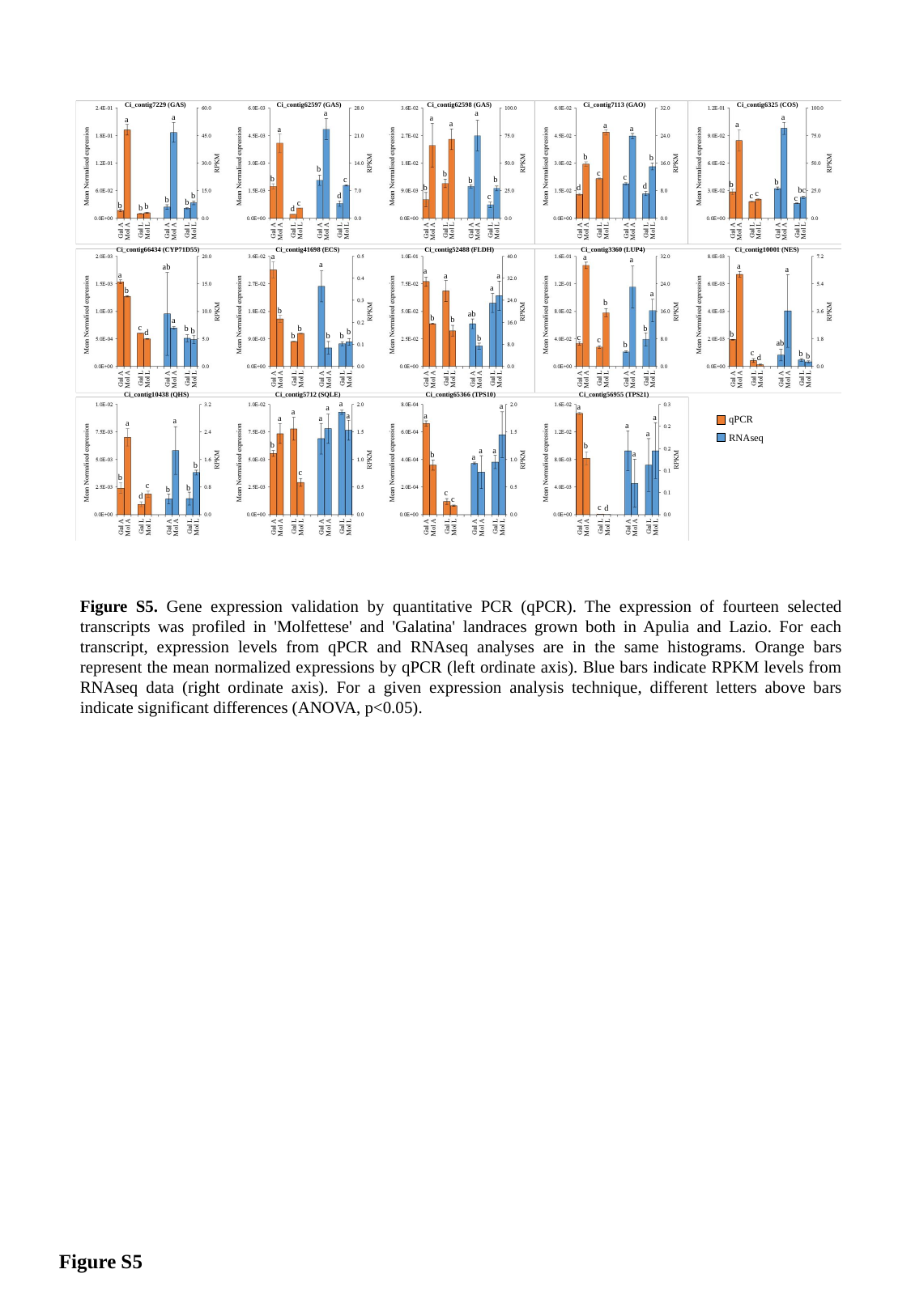

Ci_contig7229 (GAS)
Ci_contig62597 (GAS)
Ci_contig62598 (GAS)
Ci_contig7113 (GAO)
Ci_contig6325 (COS)
a
a
a
a
a
a
a
a
a
a
a
b
b
b
c
b
c
b
c
b
b
b
b
d
b
d
bc
c
d
b
c
c
c
b
b
c
b
b
b
d
Ci_contig66434 (CYP71D55)
Ci_contig41698 (ECS)
Ci_contig52488 (FLDH)
Ci_contig3360 (LUP4)
Ci_contig10001 (NES)
a
a
a
a
a
ab
a
a
a
a
a
a
b
a
b
b
ab
b
b
a
c
b
b
b
b
b
d
b
b
b
b
c
b
c
ab
b
c
b
b
d
Ci_contig10438 (QHS)
Ci_contig5712 (SQLE)
Ci_contig65366 (TPS10)
Ci_contig56955 (TPS21)
a
a
a
a
a
a
a
a
a
a
qPCR
a
a
a
a
RNAseq
b
b
a
a
a
b
a
b
c
b
c
b
b
c
d
c
c
d
Figure S5. Gene expression validation by quantitative PCR (qPCR). The expression of fourteen selected transcripts was profiled in 'Molfettese' and 'Galatina' landraces grown both in Apulia and Lazio. For each transcript, expression levels from qPCR and RNAseq analyses are in the same histograms. Orange bars represent the mean normalized expressions by qPCR (left ordinate axis). Blue bars indicate RPKM levels from RNAseq data (right ordinate axis). For a given expression analysis technique, different letters above bars indicate significant differences (ANOVA, p<0.05).
Figure S5

## Slide 6
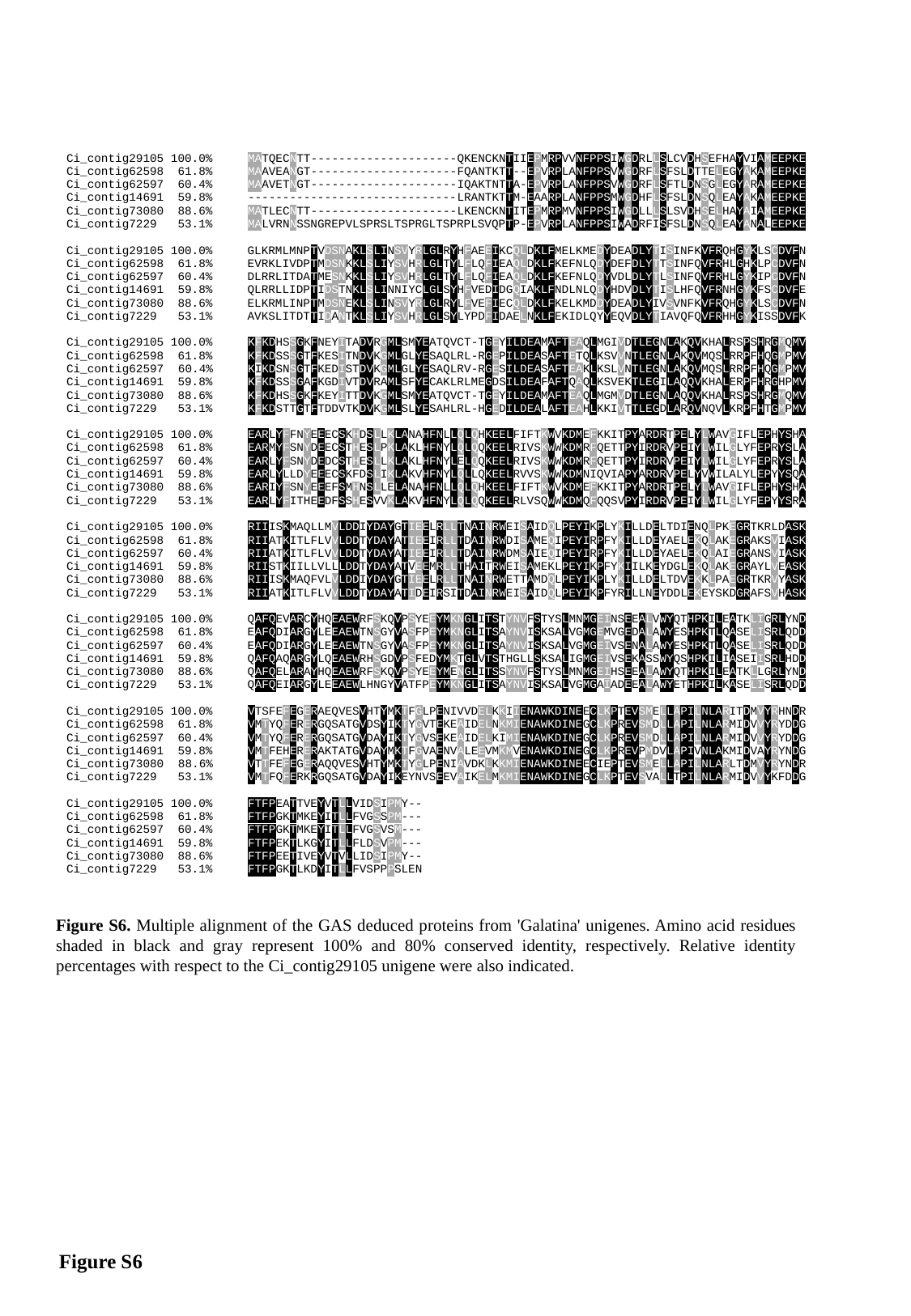

Figure S6. Multiple alignment of the GAS deduced proteins from 'Galatina' unigenes. Amino acid residues shaded in black and gray represent 100% and 80% conserved identity, respectively. Relative identity percentages with respect to the Ci_contig29105 unigene were also indicated.
Figure S6

## Slide 7
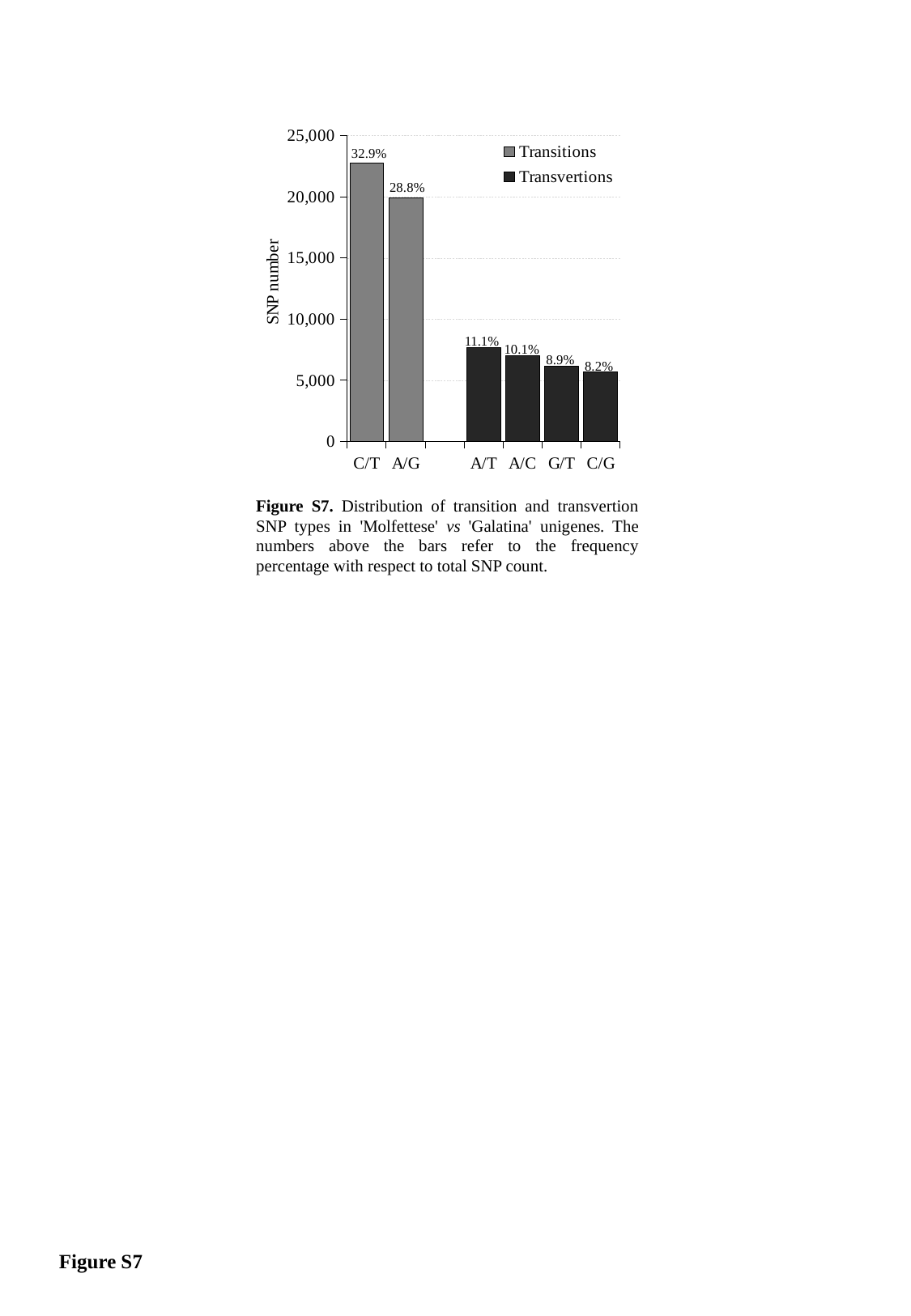

### Chart
| Category | Transitions | Transvertions |
|---|---|---|
| C/T | 22798.0 | None |
| A/G | 19944.0 | None |
| | None | None |
| A/T | None | 7691.0 |
| A/C | None | 7029.0 |
| G/T | None | 6181.0 |
| C/G | None | 5709.0 |32.9%
28.8%
11.1%
10.1%
8.9%
8.2%
Figure S7. Distribution of transition and transvertion SNP types in 'Molfettese' vs 'Galatina' unigenes. The numbers above the bars refer to the frequency percentage with respect to total SNP count.
Figure S7
